# Intracellular Pathogens: Host Immunity and Microbial Persistence Strategies

**DOI:** 10.1155/2019/1356540

**Published:** 2019-04-14

**Authors:** Aneesh Thakur, Heidi Mikkelsen, Gregers Jungersen

**Affiliations:** ^1^Department of Pharmacy, Faculty of Health and Medical Sciences, University of Copenhagen, Universitetsparken 2, 2100 Copenhagen Ø, Denmark; ^2^Section for Immunology and Vaccinology, National Veterinary Institute, Technical University of Denmark, Kemitorvet, 2800 Kgs. Lyngby, Denmark; ^3^Section for Protein Science and Biotherapeutics, Department of Biotechnology and Biomedicine, Technical University of Denmark, Kemitorvet, 2800 Kgs. Lyngby, Denmark

## Abstract

Infectious diseases caused by pathogens including viruses, bacteria, fungi, and parasites are ranked as the second leading cause of death worldwide by the World Health Organization. Despite tremendous improvements in global public health since 1950, a number of challenges remain to either prevent or eradicate infectious diseases. Many pathogens can cause acute infections that are effectively cleared by the host immunity, but a subcategory of these pathogens called “intracellular pathogens” can establish persistent and sometimes lifelong infections. Several of these intracellular pathogens manage to evade the host immune monitoring and cause disease by replicating inside the host cells. These pathogens have evolved diverse immune escape strategies and overcome immune responses by residing and multiplying inside host immune cells, primarily macrophages. While these intracellular pathogens that cause persistent infections are phylogenetically diverse and engage in diverse immune evasion and persistence strategies, they share common pathogen type-specific mechanisms during host-pathogen interaction inside host cells. Likewise, the host immune system is also equipped with a diverse range of effector functions to fight against the establishment of pathogen persistence and subsequent host damage. This article provides an overview of the immune effector functions used by the host to counter pathogens and various persistence strategies used by intracellular pathogens to counter host immunity, which enables their extended period of colonization in the host. The improved understanding of persistent intracellular pathogen-derived infections will contribute to develop improved disease diagnostics, therapeutics, and prophylactics.

## 1. Introduction

Infectious diseases caused by bacteria, viruses, fungi, and parasites can be categorized into extracellular or intracellular pathogens from an immunopathological perspective. Most encounters with these pathogenic agents lead to an acute infection, followed by the development of clinical signs. These infections are relatively brief, and in a healthy host, following onset of appropriate immune response, the infection subsides with elimination of involved pathogens within days. Acute infections are the typical, expected course for bacteria like *Streptococcus pneumonia* and *Haemophilus influenzae*, both commensals of the nasal cavity or viruses like influenza virus and rhinovirus. However, some pathogens can evade elimination by the host immune system using various mechanisms and cause persistent infections, which might lead to lifelong, latent infections. Unlike an acute infection, a persistent infection is not cleared quickly and the pathogen, pathogen genome, or pathogen-derived proteins continue to be produced for long periods; e.g., an infectious Lymphocytic choriomeningitis virus or *Salmonella* Typhi bacteria may be produced continuously or intermittently for months or years [1]. Commensal microorganisms, which reside at mucosal surfaces, form a protective barrier that shields the host from microbial invaders [2]. A compromised immune system, an altered microbiota, or breached skin or mucosal barriers allow these microorganisms the opportunity to cause infections. Their ability to persist and to be transmitted without detection gives such opportunistic pathogens a unique disease biology that warrants special attention [3]. Persistent infections can be classified into chronic infections, if they are eventually cleared from the host and latent or slow infections, if they last the life of the host. In chronic infections, there is a high level of replication or high burden of the pathogen during the pathogen persistence, e.g., chronic *Salmonella* Typhi infection. In a latent infection, an initial acute infection is followed by a dormant phase and repeated spells of reactivation, which mostly results in the production of infectious agents but may or may not be accompanied by symptoms. Examples of latent viral infections include Herpes Simplex Virus (HSV) and Epstein-Barr Virus (EBV), while latent bacteria include *Mycobacterium tuberculosis* and syphilis causing *Treponema pallidum*. In slow infections, a number of years intercede from the time of initial contact of the infectious agent, mostly viruses, until the appearance of noticeable symptoms, e.g., human immunodeficiency virus (HIV) and in rare cases subacute sclerosing panencephalitis caused by measles virus [4], which normally is an acute infection. Intracellular pathogens can adopt one of these different patterns of infection in the host. Interestingly, many intracellular pathogens thrive inside one of the most efficient cell types of antimicrobial defense, namely, mononuclear phagocytes such as macrophages and dendritic cells (DCs) [5]. Alternatively, the endosomal compartment or the cytosol of host cells such as neutrophils, fibroblasts, or epithelial cells serves as important habitat for intracellular pathogens [5, 6]. By adopting this intracellular lifestyle, the pathogens gain access to otherwise restricted nutrient sources and enjoy rare competition from other microbes [5]. In addition, their intracellular habitat protects them from direct attack by antibodies. Once inside the host cell, a pathogen must replicate without killing the host cell hastily and without disturbing host cell function and integrity to ensure its own prolonged survival. Over millions of years of coevolution with their hosts, pathogens have evolved various strategies for symbiosis and to evade killing by the host immune system [7]. These evasion strategies of microbes have improved our knowledge of infection biology to a great deal for the development of suitable therapeutics and vaccines. Furthermore, it has contributed immensely to understanding of host-pathogen interactions in many persistent infections constituting a great burden of morbidity and mortality in human diseases.

In this review, we discuss various host-induced immune mechanisms that are involved in the mediation of protection against microbial infections, and we address the current understanding of persistent intracellular infections, including mechanisms of their persistence and host-pathogen interaction.

## 2. Host Defense against Microbial Infections

**Figure 1:**
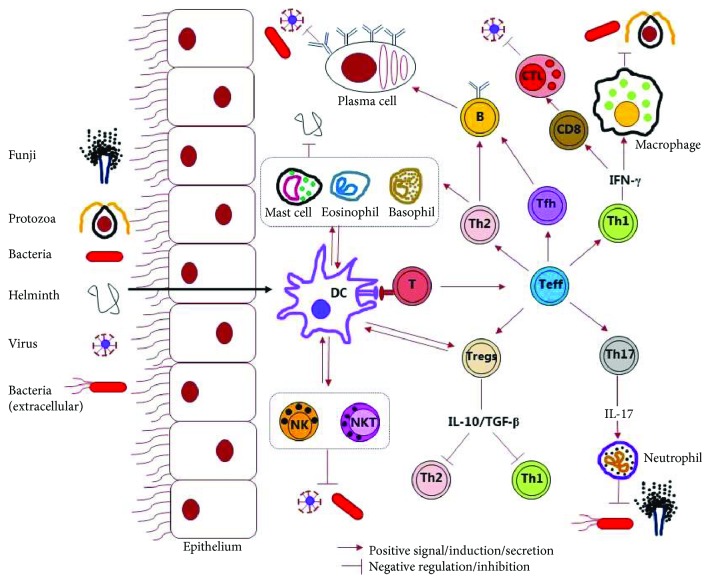
Schematic representation of the host immune response against microbial pathogens. Microbial pathogens or antigens can be taken up by the antigen-presenting cells, mostly dendritic cells (DCs), once they breach the epithelial barrier. Antigens are presented to the naive T cells by the activated DCs through major histocompatibility complex–T cell receptor interaction, which leads to activation and expansion of antigen-specific effector T cells (Teff). Teff differentiate into one of the different subtypes, e.g., helper T cells (Th)1, Th2, follicular helper T cells (Tfh), Th17, or regulatory T cells (Tregs), depending on the cytokine milieu of the microenvironment. Th1 cells activate macrophages or CD8^+^ T cells through production of IFN-*γ*. Activated macrophages fuse their lysosomes more efficiently to phagosomes, exposing intracellular microbes to a variety of microbicidal lysosomal enzymes and toxic oxygen and nitrogen metabolites. Cytotoxic T cells (CTL) destroy pathogens through release of perforins and granzymes or induce apoptosis of infected cells. Th2 and Tfh cells activate B cells through production of cytokines and induce the differentiation of B cells into plasma cells, antibody class switching, and affinity maturation of antibodies, which remove the pathogen by neutralization, opsonization, and phagocytosis. Th17 cells participate in neutrophil activation and immune regulation by producing cytokine IL-17A, which is required for protection against extracellular and some intracellular pathogens. Tregs regulate immune responses to pathogens and maintain self-tolerance by negatively regulating Th1 and Th2 cells, e.g., by producing cytokines IL-10 and TGF-*β*. Innate immune cells such as eosinophils, basophils, and mast cells play an important role in protection against parasitic infections including helminth infections. Natural killer (NK) and natural killer T (NKT) cells, which form a bridge between innate and adaptive immunity, also contribute to antibacterial and antiviral immunity. NK cells have similar functions as the CTL while NKT cells produce cytokines to execute their killing functions.

Intracellular persistent infections change the nature of the host, alter immune function and immunological protection, and predispose the host to other persistent infections [1]. The immune system is an extraordinary diverse compilation of cells that comprise the two arms of the immune system, namely, innate and adaptive. Innate and adaptive immune systems are linked, and innate immune recognition controls activation of adaptive immune responses [8]. The innate immune system constitutes the first line of host defense against pathogens and recognizes evolutionary conserved repetitive molecules on pathogens, named pathogen-associated molecular patterns through germline-encoded pattern recognition receptors (PRRs) such as Toll-like receptors (TLR), C-type lectin receptors, nucleotide-binding oligomerization domain- (NOD-) like receptors, and retinoic acid-inducible gene- (RIG-) I-like receptors [9]. Innate immune defenses are mediated by complement proteins, phagocytic cells (monocytes, macrophages, and neutrophils), and natural killer (NK) cells, and the effector mechanisms of these cells do not induce immunological memory. Adaptive immunity is comprised of cell-mediated and humoral branches and has a broader and fine-tuned repertoire of recognition due to antigen variability and frequent mutations. The key features of the adaptive immune system are the immune effector functions, which are pathogen-specific owing to receptor rearrangement mechanisms such as somatic hypermutation (B cell receptor) and V(D) J recombination (both T and B cell receptor), immunological memory, and the regulation of host immune homeostasis and tolerance. In recent years, the accumulating scientific evidence shows that after infection or vaccination, innate immune cells such as monocytes, macrophages, or NK cells remember a previous exposure to microbial pathogens or antigens and undergo long-term functional and epigenetic reprogramming [10, 11]. These changes, described as “trained immunity,” lead to increased responsiveness during secondary infection, increased production of inflammatory mediators, and increased capacity of protection against infection through mechanisms independent of T or B cell adaptive responses. Although the specificity and the immunological memory of innate immune cells cannot match with the highly sophisticated adaptive immune response, the contribution of trained immunity to host defense against infection should not be underestimated. The concept of trained immunity has potential application for developing improved vaccines [12, 13] as well as modulation of adverse effects of inflammatory diseases [14].  Figure 1 gives an overview of key host immune responses against microbial pathogens.

### 2.1. Cell-Mediated Immunity in Microbial Infections

All immune responses are driven by T lymphocytes maturing in the thymus and B lymphocytes maturing in the follicles of secondary lymphoid tissues such as spleen and lymph nodes. Both lymphocyte lineages follow almost similar stages of development and activation; however, there is a remarkable diversity of effector functions. The various lymphocyte subsets display a large variation of cell surface signaling molecules, which are vital for differentiation, recognition, and cellular functions [15]. Activation of antigen-specific T cells is a complex process and requires the help of antigen-presenting cells (APCs). Once activated, T cells can differentiate into distinct subsets and execute their effector functions (Table 1). While antibodies (produced by B cells matured into plasma cells, see Section 2.2) have the possibility to neutralize extracellular functions of microbial-derived molecules, cell-mediated immunity relies on the various T cells responding to the presence and presentation of microbial-derived molecules, typically peptides, and is unable to block the function of the antigenic molecule.

#### 2.1.1. Intracellular Effector/Killing Mechanisms

Professional phagocytes, such as macrophages, neutrophils, and dendritic cells, recognize and internalize microorganisms through recognition by PRRs or by opsonizing antibodies binding to Fc*γ* receptors. This leads to a cascade of signaling events, remodeling, and focal exocytosis of endomembranes forming a phagosome. Maturation of the phagosome is characterized by changes in acidity and acquisition of GTPases, proteases, and other acid hydrolases and occurs through stages of early and late phagosome and the highly acidic phagolysosome formation [114]. Microbicidal activity of the phagolysosome can be attributed to acidification, reactive toxic oxygen species (ROS), reactive nitrogen intermediates (RNI), antimicrobial proteins, and peptides [115]. Antimicrobial proteins include secretory granules like lactoferrin, which interfere with the iron metabolism [116], while a membrane protein, natural resistance-associated macrophage protein 1, exerts bacteriostatic effects by extruding Fe^2+^, Zn^2+^, and Mn^2+^ from the phagosomal lumen [117]. Antimicrobial peptides include defensins, cathelicidins, lysozymes, lipases, and proteases [114]. Microbial degradation by lysosomal enzymes may also lead to generation of antigenic peptides suitable for presentation by MHC class II molecules and subsequent CD4^+^ T cell activation.

#### 2.1.2. Proinflammatory Cytokines

IFN-*γ* is a type II interferon and a key cytokine in intracellular infections that orchestrates many distinct cellular programs and signaling events resulting in heightened immune surveillance and immune function. IFN-*γ* coordinates a shift from innate to adaptive immunity through mechanisms such as promoting development of a Th1-type response by inducing IL-12 and IL-18 production [118], B cell isotype switching to IgG2a [119], and regulating leukocyte trafficking. IFN-*γ* also upregulates expression of MHC class I and class II molecules and promotes induction of cell-mediated immunity and activation of Th1 cells [120]. Autophagy has been recognized as a key mechanism by which IFN-*γ* exerts control over intracellular pathogens such as *M. tuberculosis* [121], *Toxoplasma gondii* [122], *Chlamydia trachomatis* [123], *Salmonella* [124], and *Listeria monocytogenes* [125]. The crucial role of IFN-*γ* in clearing intracellular infections has been demonstrated using either antibody-mediated neutralization assays, IFN-*γ* receptor *α* chain, or IFN-*γ* gene knockout (KO) mice for infections with *M. tuberculosis* [16], *Chlamydia* [17], *Plasmodium* [18], *Francisella tularensis* [19], *Leishmania* [20], and *Rickettsia* spp. [21]. Moreover, IFN-*γ* therapy was found to improve the outcome of disease status in tuberculosis patients [126]. In addition to CD4^+^ Th1 as the principle source of IFN-*γ*, CD8^+^ T cells also contribute to IFN-*γ* secretion in *M. tuberculosis* [127], *Chlamydia* [128], *L. monocytogenes* [129], *Rickettsia* [21], and *F. tularensis* [71] infections. In viral infections, in addition to various effector mechanisms, IFN-*γ* also induce antiviral enzymes such as protein kinase dsRNA-regulated (PKR), dsRNA-specific adenosine deaminase, and guanylate-binding proteins as well as the enzymes involved in proapoptotic effects including PKR, death-associated proteins, FAS/FAS ligand, cathepsin D, and caspase 1 [120]. Despite the role of IFN-*γ* in protection against many intracellular infections, it was shown recently that protection mediated by CD4^+^ memory T cells from *L. monocytogenes* was mostly dependent on TNF-*α*, whereas IFN-*γ* was found to play only a minor role [130]. Also, studies with tuberculosis (TB) infection suggest an alternative mechanism of protection other than IFN-*γ* [131, 132]. These findings emphasize that although IFN-*γ* is important for protection against various intracellular pathogens, this cytokine alone is not sufficient as a marker of protection [133]. Besides IFN-*γ*, TNF-*α* also activates macrophages and adopts similar killing strategies against pathogens including inducible nitric oxide synthase (iNOS), ROS, RNI, and autophagy. Moreover, TNF-*α* has a key role in granuloma formation and containment of disease in TB [134]. Similar to IFN-*γ*, studies using KO mouse models deficient in either TNF-*α* or p55 TNF-*α* receptor have defined a central function for this cytokine in many intracellular bacterial infections such as *M. tuberculosis* [29], *Salmonella* [22], *Chlamydia* [23], *Brucella* [24], *L. monocytogenes* [25], and *F. tularensis* [26], and in viral infections such as HSV [27] and HIV [28]. However, in studies on *Plasmodium* infection, contradictory results were obtained regarding the protective role of TNF-*α* in malaria [135, 136].

Other proinflammatory cytokines that are involved in the defense against intracellular pathogens to various degrees are interleukin- (IL-) 1*α*, IL-2, IL-6, IL-8, IL-12, and IL-18. The cytokines IL-1*α*, IL-6, and IL-8 play a key role in innate response and macrophage activation during persistent intracellular infections such as in *Mycobacterium* [137], *Chlamydia* [138], *Leishmania* [139], *Listeria* [140], and HIV infection [141]. IL-1*α* also potentiates IL-12-mediated induction of IFN-*γ* from NK cells during intracellular infections. IL-2 production in intracellular infections is associated with stimulation of cytotoxic T cells and differentiation as well as development of T cell immunological memory [142, 143]. IL-2 is involved in the maturation of regulatory T cells (Tregs), and IL-2 deprivation is associated with transient reduction in Tregs, which is essential for optimal T cell responses and host resistance to microbial pathogens [144]. IL-12 and IL-18 are key cytokines regulating IFN-*γ* production during infection and serve as a bridge connecting innate and adaptive immunity [145]. IL-18 maturation and release is promoted by caspase-1, a central mediator of innate immunity that in turn is activated by a multiprotein oligomer, termed the inflammasome [146]. The inflammasome is a molecular complex, which is involved in the activation of inflammatory caspases; promotes the maturation and secretion of proinflammatory cytokines, IL-1*β*, and IL-18; and activates inflammatory responses [147]. IL-12 and IL-18 in combination further increase IFN-*γ* levels from macrophages, NK cells, and T cells and thus are important cytokines in many persistent intracellular infections [30–35].

#### 2.1.3. Conventional, Regulatory, and Unconventional T Cells

T lymphocytes that express an *αβ* TCR as well as a coreceptor CD4 or CD8, i.e., the so-called conventional T cells recognizing antigens presented in a peptide-MHC complex, have a central role in protective and aberrant immunity against persistent intracellular infections. There are many subsets of CD4^+^ T cells, such as T-helper 1 (Th1), Th2, Th17, follicular helper (Tfh), and regulatory T cells (Tregs), and all these subsets cooperate or interfere with each other to control infection (Table 1). A CD4^+^ Th1 cell response is considered to have a protective role against *M. tuberculosis* infection due to production of cytokines such as IFN-*γ* or TNF-*α*, which recruit and activate innate immune cells, like monocytes and granulocytes [16, 29]. Th1 cells also play an important role in protective immunity against other persistent intracellular infections [17–20]. Th17 cells have been found to be induced following infections with *M. tuberculosis* [37], *M. bovis* [40], *Salmonella enterica* [83], *F. tularensis* [41], *L. monocytogenes* [50], Leishmaniasis [148], and many viral infections such as influenza [43], hepatitis B virus (HBV) [149], and HIV [150]. The IL-23/Th17 pathway was found to mediate inflammatory responses in intracellular pathogens, but was not critical for protection against disease as IL-17RKO and IL-23KO mice were not found to be more susceptible to infection with *M. tuberculosis* [37, 151] or *S. enterica* [36] compared to the wild type. However, other mouse studies show that absence of the IL-23/Th17 pathway increases susceptibility to *F. tularensis* [152], *Chlamydia muridarum* [39], and *M. bovis* bacillus Calmette–Guérin [38]. Similarly, IL-17RKO and IL-23KO mice have reduced neutrophil recruiting chemokines such as CXCL-1, -2, -5, and -8 in the liver and were more susceptible to *L. monocytogenes* infection [50, 153]. In HBV patients, Th17 cell frequency was associated with disease progression and liver injury [149]. However, increased frequencies of IL-17/IL-22 cells were observed in chronic HBV patients but without IL-17 correlation with liver fibrosis [154]. Th17 cells have been found to be involved in the disease progression and pathogenesis in HIV and Simian immunodeficiency virus infections by influencing innate immune response and limiting chronic inflammation [150]. Thus, Th17 cells have diverse roles spanning from cell-mediated direct protective and indirect helper effects, which are important for intracellular immunity. CD8^+^ T cells or CTLs remove cells infected with intracellular pathogens as well as cancerous cells through contact-dependent lysis and release of cytokines. It is well-known that CTLs are critical for clearance of many viral infections, but their exhaustion during chronic viral infections is accompanied with impaired function and poor survival [155, 156]. Various studies suggest that IFN-*γ* production by CTLs is required for the clearance of intracellular bacterial infections such as *M. tuberculosis* [68], *C. trachomatis* [69], *L. monocytogenes* [70], *Brucella* [67], *T. gondii* [157], *F. tularensis* [158], and *Rickettsia* [66]. Likewise, perforin [73–75] and granzyme [75, 76] deficiency has been associated with increased disease pathology in chronic infections with viruses, bacteria, and parasites.

CD4^+^FoxP3^+^CD25^+^ and CD8^+^ Tregs play a critical role in maintaining immunological tolerance to self-antigens and in suppressing excessive immune responses deleterious to the host. As an example, CD4^+^ Tregs were isolated and correlated with apoptotic activity from human lepromatous leprosy patients [159]. In addition, patients with active TB were found to have increased frequencies of CD4^+^ Tregs producing IL-10 [56]. In a mouse model of *Leishmania donovani* infection, CD4^+^Foxp3^+^ Tregs play an important role in delaying the development of splenic pathology and restricting leukocyte expansion [57]. In malaria, Tregs impede host-mediated protective immunity through CTL-associated protein-4 (CTLA-4) that delays parasite clearance [58]. Similarly, increased numbers of circulating CD4^+^ Tregs have been described in viral infections such as human cytomegalovirus (HCMV) and hepatitis C virus (HCV) [160]. In TB [59] and HCV [60], HCMV [161], and EBV [61] infections, CD8^+^ Tregs induction inhibits effector T cell responses and pathogen clearance chiefly through TGF-*β*.

Another category of T cells, the so-called unconventional T cells, have been identified in persistent intracellular infections. These T cells are non-MHC-restricted T cells, which recognize nonpolymorphic antigen-presenting molecules and have a more limited TCR repertoire. The unconventional T cells include *γδ* T cells, NK cells, NKT cells, invariant NKT (iNKT) cells, and mucosal-associated invariant T cells (MAIT) cells. *γδ* T cells have increasingly been identified to play an important role in host defense against persistent intracellular infections and serves as a bridge between innate and adaptive immunity [162]. *γδ* T cell response to infection is staged and may occur before or after involvement of *αβ* T cells. *γδ* T cells in these immune stages perform different functions due to differential production of Th1 (early stage)/Th2 (late stage) cytokines, which has been observed in infections with influenza A [163], *Schistosoma mansoni* [164], and *L. monocytogenes* [82]. Additionally, *γδ* TCR-deficient mice were found to have 100% mortality following *Nocardia asteroides* intranasal challenge due to poor neutrophilic infiltration in the lungs, which could be caused by decreased IL-17 production [77]. Depletion of IL-17A-producing *γδ* T cells resulted in increased bacterial growth due to poor generation of antigen-specific CTL responses [82]. Similarly, increased susceptibility to *B. abortus* infection was observed on depletion of *γδ* T cells in mice compared to wild types [78]. In advanced stages of *L. monocytogenes* infection, depletion of *γδ* T cells was characterized by liver necrosis, secondary inflammation, and disruption of macrophage homeostasis mediated by TNF-*α*^+^CD8^+^ T cells and reduced IL-10 [79] and IL-17 [82] production by *γδ* T cells. Functional loss of *γδ* T cells as a result of upregulation of the FAS and FAS ligand has been correlated with disease progression in *M. tuberculosis* [80] and HIV-1 infection [81]. Thus, the role of *γδ* T cells in persistent intracellular infections appears to be a regulation of inflammation and subsequent pathogen elimination. NK cells are cytotoxic lymphocytes and are important connectors between innate and adaptive immunity *via* production of cytokines and interaction with APCs [165]. The role of NK cells has been documented in the control of tumors and parasitic and early viral infections. Defects in NK cell activity, such as decreased production of IFN-*γ* or cytotoxicity, have been associated with many viral infections [85, 86]. In the case of HIV infection, NK cell number and function decrease with disease progression [166]. A role for NK cells has been identified in many protozoal infections including leishmaniasis and malaria [167]. NK cell-derived IFN-*γ* differentially regulates innate resistance in mice infected with intracellular pathogens [87, 88]. Despite the redundant functions of NK cells in several conditions, NK cells also act as regulatory cells during inflammation and influence adaptive immune responses [165]. NKT cells have an immunoregulatory function promoting cell-mediated immunity to infectious pathogens as well as tumors. In intracellular infections, iNKT cells are characterized by release of cytokines such as IFN-*γ*, TNF-*α*, IL-4, IL-5, IL-13, IL-17, chemokines, and rapid effector functions as in *Salmonella*, *Ehrlichia*, *M. tuberculosis*, *Trypanosoma cruzi*, and many viral infections [168]. A significant impairment of iNKT cells has been reported in chronic HIV type 1 infection [90]. In influenza A virus infection, IL-22 production by iNKT cells was involved in control of lung epithelial damage but had no direct effect on viral replication [91]. In chronic HBV patients, however, restoring the number of circulating iNKT cells resulted in control of viral replication accompanied with higher expression of CCR5 and CCR6 [92]. Contrary to these positive effects, iNKT cells were found to have a detrimental role in the pathology following experimental dengue virus infection in mice [93]. Distinct iNKT cell subsets are induced during intracellular bacterial infections leading to differential adaptive immune responses and control of infection as has been observed in *Chlamydophila pneumoniae* infection displayed by IFN-*γ* production by iNKT cells and by IL-4 production in *C. muridarum* infection [94]. In *M. tuberculosis* infection, increased CD8+ iNKTs were correlated with favorable disease outcome post-BCG vaccination [95]. A role for MAIT cells in immune protection against intracellular infections has been demonstrated, which is consistent with the pathogens sharing the riboflavin pathway and producing riboflavin-derived antigens. In *M. tuberculosis* infection, MAIT cell levels are reduced in peripheral blood and lungs of patients with active pulmonary TB [169]. Similarly, in HCV [170], HBV [171], and HIV [172] infection, MAIT cells are depleted from the blood. This depletion was accompanied with expression of tissue homing markers and detection of MAIT cells in affected tissues, which suggests that these cells are recruited to the sites of infection. The depletion of MAIT cells in mice impedes protection against *M. tuberculosis* [96], *F. tularensis* [98], *S. enterica* [101], *H. pylori* [100], *Legionella* spp. [99], and influenza virus [97] elucidating their role in protective immunity.

Both conventional and unconventional T cells complement each other during host immune responses against persistent intracellular infections. While conventional T cells mostly mediate antigen-specific functions and immunological memory of the cell-mediated immunity, unconventional T cells have a limited TCR diversity but respond very rapidly to pathogenic assaults. A full spectrum of cell-mediated immune responses encompassing conventional, unconventional, and regulatory T cells determines the immunological outcome in persistent intracellular infections where the evolution of pathogens has led to diverse escape mechanisms to establish persistence in the host.

### 2.2. Humoral Immunity in Microbial Infections

Humoral immunity is mediated through antibodies produced by B lymphocytes, which are also APCs, matured into plasma cells. B cells and antibodies contribute significantly to shape the immune response to and/or induce protection against many persistent intracellular pathogens [104, 105, 173] with the important distinction from cell-mediated immunity, that antibodies may functionally block the antigenic target. B cells undergo class switching and affinity maturation in the germinal centers to form antibodies of isotypes such as IgG, IgA, and IgE, which mediate their protective effects *via* neutralization, opsonization, and complement activation. Neutralization by antibodies is an important classical effector mechanism against viruses [174] and is a key correlate of protection for many infections [175]. Recently identified nonclassical antibody functions include direct antimicrobial activity, alteration of signaling by engaging F_C_R, immunomodulation, and modulation of microbial physiology [176]. Previously, it was believed that immunoglobulins could not enter infected cells and thus do not participate in combating intracellular bacterial infections. However, in *L. monocytogenes* infection, the anti-listeriolysin O antibody neutralizes listeriolysin toxin and protects the host from infection [177]. A comparison between the antibody profiles of latently versus actively *M. tuberculosis* infected individuals also points to a functional role of antibodies in the control of TB [106], and naturally occurring IgM from B1 cells have been reported to induce innate disease resistance against intracellular infection with influenza virus in mouse models [178]. In addition to the antigen specificity of antibodies, the different Fc variations may also have both pro- and anti-inflammatory functions and enhance microbial clearance through complement activation or idiotype-anti-idiotype interactions [176]. The cellular basis for these properties of antibodies is associated with ligation to stimulatory and inhibitory F_C_Rs [179]. In line with this, F_C_Rs were shown to be key elements in protective responses against intracellular pathogens chiefly through oxidative burst, antibody-dependent cellular cytotoxicity, and induction of T cell responses by cytokines for infections with *M. tuberculosis* [106], *C. trachomatis* [102], *S. typhimurium* [180], *F. tularensis* [107], *Leishmania major* [103], *Legionella pneumophila* [104], *L. monocytogenes* [108], and *T. gondii* [105]. A complete T cell independent humoral immune response mediated by B cells and antibodies was even demonstrated in *Ehrlichia muris* infection [173]. In addition, low secreted IgA (sIgA) was associated with disease pathology in polymeric-Ig receptor-deficient mice [109–113], highlighting the role of sIgA in protection against persistent pathogens. In chronic intracellular infections, the same antibody may be proinflammatory or anti-inflammatory depending on the host and the stage of infection; e.g., during *Cryptococcus neoformans* infection, administration of IgG1 before or after the onset of infection can result in anti- or proinflammatory effect, respectively [176]. It thus appears that the protection mediated by antibodies cannot be defined solely by molecular structure and glycosylation of antibodies but also depends on components of host as well as the pathogen and the stage of infection [176].

## 3. Mechanisms of Microbial Persistence

**Figure 2:**
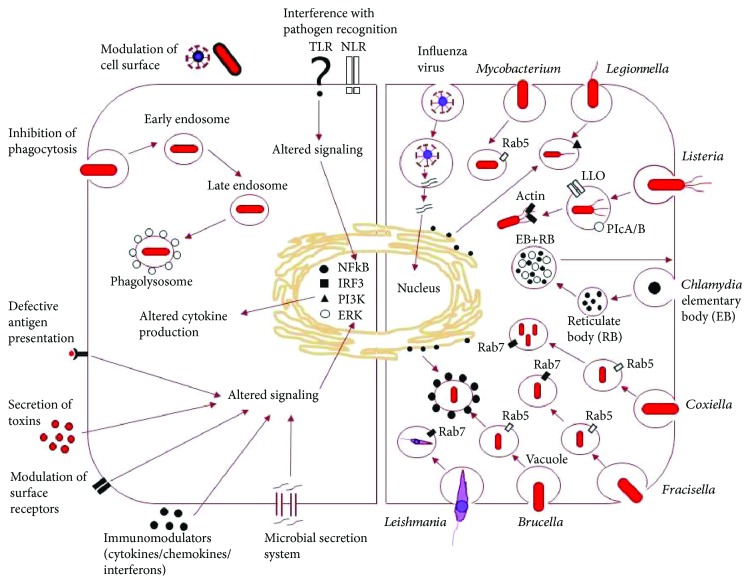
Schematic representation of the mechanisms of persistence of selected intracellular pathogens. Left: an overview of the various mechanisms used by pathogens to overcome innate and adaptive immune responses. The major strategies are discussed in more detail in the text. Right: evasion strategies of various phagocytic mechanisms by selected intracellular pathogens. Viruses such as influenza virus are able to inhibit the activation of antiviral mechanisms, such as the production of interferon upon viral infection, and enter the nucleus. *Mycobacterium tuberculosis* after phagocytosis acquires the early endosome marker Rab5, which blocks fusion with the lysosome, and the mycobacteria replicate in this early endosome. *Legionella pneumophila* resides and multiplies in vacuoles that acquire Rab1 and secretes effector molecules *via* its type IV secretion system, which inhibits phagolysosome formation. *Listeria monocytogenes*-engulfed phagosome undergoes acidification, which perforates the phagosomal membrane and the bacteria escape into the cytosol, where they move within and then among cells with actin polymerization. *Chlamydia* spp. are present as nonreplicating infectious “elementary body” and intracytoplasmic replicating noninfectious “reticulate body.” The elementary body induces its own endocytosis upon exposure to host cells and survives and multiplies inside phagolysosome before infecting the new host. *Coxiella burnetii* and *Brucella abortus* are present inside a vacuole, which becomes acidic and acquires Rab5 followed by Rab7 that prevents phagolysosome formation. The *Francisella tularensis* phagosome acquires Rab5 (early endosome) and then Rab7 (late endosome). Late endosome is not acidified, which disrupts the phagosomal membrane discharging the bacteria into the cytosol. These vacuoles fuse with the endoplasmic reticulum, which allow bacterial replication. *Leishmania* spp. phagosome becomes acidic phagolysosome, which bears Rab7, and the parasite survives and multiplies inside the phagolysosome.

One characteristic of intracellular pathogens is their ability to maintain infection in the host even in the presence of innate and adaptive immune responses [181]. In some cases, persistent intracellular infections are asymptomatic, although the infection can pose a risk to the host, especially if the disease is reactivated from an innocuous state of dormancy. Persistent infections can be divided into two groups. One includes those pathogens, which are kept in check by adaptive immune responses in a state of dormancy but are not completely removed from the host, such as *M. tuberculosis* [16, 182, 183] and *S. enterica* [184]. The second group includes opportunistic pathogens that reside among commensal flora in the mucosa without inducing adaptive immune responses in healthy hosts, but are capable of establishing active and threatening infection in immunocompromised hosts, such as *Neisseria* [185]. Thus, there is always an intimate crosstalk between the host and the pathogen, and the pathogens have evolved numerous anti-immune strategies for continuous lifelong survival to escape host immune elimination by overcoming both innate and adaptive immunity [181]. This balance of host immune response and pathogen counter-defense contributes to the complexity of persistent infections.  Figure 2 summarizes the mechanisms of persistence of selected intracellular pathogens.

Despite the diversity, there are several general mechanisms for subversion of host immune responses that are shared between microbial pathogens. These can be divided into two broad groups: (a) evasion of host immune recognition such as modulation of microbial surfaces, secretion of immunomodulators, antigenic variation, and hiding in safe target cells or tissues (Table 2) and (b) modulation and suppression of host immune responses such as evasion of phagocytosis, innate immune receptors, complement system, cytokines, or chemokines; inhibition of apoptosis; resistance to host effector mechanisms; and induction of inappropriate immune responses such as immunosuppression and induction of Tregs (Table 3). Strategies adopted by persistent microbial pathogens is a broad topic, and reviewing it comprehensively is more suitable for a full book, so we have chosen to highlight some key mechanisms, which the pathogens use to ensure their prolonged survival.

### 3.1. Evasion of Host Immune Recognition

#### 3.1.1. Surface Immunomodulation

The external surface of microbial pathogens is the first interface of pathogen and host interactions. This interface provides numerous opportunities for both pathogen and host to modulate and shift the immune equilibrium in their favor. Pathogens avoid immune detection by secreting immunomodulators from infected cells, including proteins and toxins [233, 234], and express receptors and inhibitors, modifying their own surface molecules/ligands [235]. Some viruses have evolved viral cell-surface proteins that mimic the structure as well as function of host cell receptors; e.g., herpes and poxviruses encode over 40 viral proteins that hijack transmembrane G-protein coupled-receptor signaling networks of the host [189, 190]. Bacterial pathogens have evolved ways to alter the TLR agonists on their surfaces such as lipid A, flagella, and peptidoglycan [236]. Many bacterial pathogens modify lipid A to avoid TLR4 detection and include *Salmonella* [186], *Neisseria* [237], and *Yersinia* [238]. In addition, some bacterial pathogens have evolved methods to avoid processing of peptidoglycan-derived muropeptides and their detection by the cytosolic receptors, NOD1 and NOD2 proteins [187]. Peptidoglycan plays an important role in the pathogenesis of many persistent intracellular infections [188].

#### 3.1.2. Secretion of Immunomodulators

Persistent bacterial pathogens have developed a secretion system to deliver virulence factors such as toxins and effectors interfering with apoptosis into the host cell, thereby enhancing intracellular survival. Out of seven such secretion systems, type III (T3SS) (used by *Chlamydia trachomatis and Salmonella typhimurium)* and type IV secretion systems (T4SS) (used by *Legionella and Brucella*) are the most widely studied [192, 193, 239]. *M. tuberculosis* uses a specialized secretion system, Esx secretion systems (ESX-1, ESX-3, and ESX-5), to deliver major T cell antigens ESAT-6 and CFP-10 into the host [191]. Similarly, secretion systems have been described for gram-positive bacteria, e.g., Ess system of *Staphylococcus aureus* [194] and the Yuk/Yue system of *Bacillus subtilis* [195]. Ess plays a key role in virulence of *S. aureus* allowing it to persist, establish staphylococcal abscesses, and evade the host immune response [194]. The Yuk/Yue system of *Bacillus subtilis* mediates YukE protein secretion and is homologous to Ess proteins of *S. aureus* [195]. In the case of viruses, secreted viral immunomodulators mimic a wide range of host molecules including cytokines, chemokines, interferons, and complement and inflammatory cascades [240, 241]. These secreted viral immunomodulatory proteins are excellent targets for developing novel immunotherapeutic strategies [242].

#### 3.1.3. Antigen Variation

Antigenic variation is another classical method adopted by persistent pathogens to avoid immune responses especially the adaptive immune responses. Among bacterial pathogens, *Neisseria* is one of the best examples for antigenic variation. The pathogenic *Neisseria* have three antigenically or phase-variable major surface determinants: the opacity (Opa) outer membrane proteins, which govern bacterial adhesion and uptake into host cells; lipooligosaccharide (LOS), which is present in the outer membrane and is involved in host interactions; and type IV pilus (Tfp), which is involved in cellular adherence [197]. There are up to 11 antigenically different Opa proteins and 12 recognized LOS immunotypes that are turned on and off independently and exhibit multiple combinations [198]. Tfp antigenic variation relies on a programmed homologous recombination system to express antigenically distinct peptide sequences [197]. Variant surface glycoprotein (VSG), the major surface component of the protozoan parasite *Trypanosoma brucei*, is another example of antigenic variation. VSG exists in the blood and tissues of its mammalian host, but during an infection, some *T. brucei* parasites will switch their VSG to a new and antigenically distinct variant, which results in a typical parasitemia in the infected host [201]. Similarly, RNA viruses use antigenic variation for evading host immune responses through the mechanisms of antigenic drift and shift as seen with HCV [202], HIV [203], and influenza virus [196]. DNA viruses, both single, e.g., parvovirus [243], and double-stranded, e.g., cytomegalovirus [244], exhibit mutations to permit selective escape from the host immunity.

#### 3.1.4. Subversion of Host Defense and Hiding

Successful pathogens thwart all or most host immune defenses to remodel their intracellular habitat into a safe compartment. Once inside professional phagocytes, pathogens can still reach a stage of persistence if they manage to counter antimicrobial effector mechanisms, escape the phagolysosome, or modify their intracellular habitat into a safe niche [245]; e.g., *Yersinia pestis* uses its T3SS to inject *Yersinia* outer proteins that counter multiple signaling responses initiated by phagocytic receptors [246]. Other bacterial pathogens avoid killing after phagocytosis by three strategies: (i) escape from phagosome, (ii) prevention of phagosome-lysosome fusion, and (iii) survival inside the phagolysosome. The first evasion strategy is adopted by *Listeria* [217, 247, 248] and *Rickettsia* spp. [218]. *L. monocytogenes* is considered as the phagosomal escape artist as it uses a sophisticated effector mechanism through listeriolysin, phospholipases, and an effector protein ActA, which causes breakdown of the phagosome and escape of bacteria into the cytosol [217, 247, 248]. *M. tuberculosis* and *Salmonella* use the second strategy for persistence. *Salmonella* uses its T3SS called Spi/Ssa that exports the SPI-2 pathogenicity island-encoded SpiC protein into the host cell cytoplasm and efficiently blocks phagosome-lysosome fusion [249]. In comparison, *M. tuberculosis* uses a combined strategy by employing a range of protein and lipid effectors such as SapM, ZmpA, kinases, and lipoarabinomannan, which deplete phosphatidylinositol 3-phosphate from early phagosomes and prevent phagolysosome formation [250]. In addition, mycobacteria use ESX secretion system to prevent phagolysosomal fusion [191]. Finally, pathogens such as *Salmonella*, *Leishmania*, *Staphylococci*, and *Coxiella* can survive and even replicate inside the acidic and hydrolytic environment of the phagolysosome. *Salmonella* uses the PhoP/PhoQ regulatory system for survival [251], while *Leishmania*, *Coxiella*, and *Francisella* in addition to replication can draw nutrients at an acidic pH of the phagolysosome [252–254]. *Staphylococcus aureus* employs mechanisms such as perturbation of macrophage phagolysosome formation [255] and inhibition of neutrophil myeloperoxidase [256]. Viruses usually subvert lysis by phagocytic cells by preventing iNOS induction, which is under the control of NF-*κ*B and STAT-1 [257]. A range of virus-encoded proteins have been identified that inhibit NF-*κ*B activation or kinases [257]. However, some viruses maintain a balance between NF-*κ*B activation and suppression to maintain a state of latency, e.g., HSV [205]. Bacterial pathogens, on the other hand, use proteins of secretion systems to modulate NF-*κ*B signaling, e.g., T3SS protein YopJ in *Yersinia* [258], AvrA in *S. enterica* [219], SseL in *S. typhimurium* [259], and T6SS effectors and a heat shock protein ClpB in *Francisella tularensis* [214]. Other bacterial effector proteins, which have been identified, are CP0236 in *C. pneumoniae* [260], ChlaDub1 in *C. trachomatis* [208], LegK1 in *Legionella pneumophila* [216], and IKK in *Toxoplasma gondii* [221].

### 3.2. Modulation or Suppression of Host Immune Responses

#### 3.2.1. Subversion of Innate Immune Receptors

One of the mechanisms for subversion of host defense by pathogens is the evasion of PRR signaling. Viruses have evolved several mechanisms to avoid detection by PRRs or to inhibit the activation of PRRs and/or their downstream signaling cascades. Earlier evidence came from studies where some viruses encoded proteins to target TLR signaling, such as pox viruses through protein A52R [261] and hepatitis viruses through TRIF protein [262]. Since then, various TLRs have been shown to be involved in responses to viral infections including TLR1, -2, -3, -4, -6, -7, -8, and -9 [263]. Many RNA viruses replicate in the cytoplasm and are detected by the cytoplasmic PRRs, MDA5, and RIG-I, which are targets for viral evasion. RNA viruses such as flaviviruses, which include dengue virus and HCV, induce membrane modifications, which prevent their recognition by RIG-I and MDA5 and result in poor induction of type I IFN [264, 265], while enteroviruses including poliovirus cleave RIG-I and MDA5 by proteases, 2A^pro^ and 3C^pro^, are required for viral polyprotein processing [266]. Influenza virus targets host TRIM25 and RIPLET proteins, which are required for the full activation of RIG-I [267]. DNA viruses replicate within the nucleus and are detected in the nucleus or in the cytoplasm by IFI16 or cGAS, respectively. In response, DNA viruses have evolved various strategies to evade these receptors; e.g., HSV-1 produces a protein, ICPo, that ubiquitinates IFI16 and results in its degradation by the ubiquitin proteasome and eventually loss of IFN induction [268]. In HIV-1 infection, the viral cDNA is protected within the viral capsid, which prevents its exposure to cGAS in the cytoplasm [269]. In addition to above, viruses also use other strategies such as targeting adaptor proteins and their kinases during downstream signaling of antiviral innate immune pathways, inhibiting transcription factors involved in IFN induction, and evading IFN-stimulated genes [270]. Among bacterial pathogens, there are only a few, which directly inhibit the PRR signaling. *Yersinia* pestis is a typical example, where the virulence antigen, LcrV, specifically hijacks the TLR2/6 pathway to stimulate IL-10 production, which blocks host protective inflammatory responses [271]. Some bacterial pathogens target intracellular signal transduction pathways such as the mitogen-activated protein kinase (MAPK) signaling axis, TGF-*β*-activated kinase 1 (TAK1), and the NF-*κ*B pathway. The effector protein YopJ of *Y. pestis* targets several MAPK and TAK1 [272]. Similarly, *Salmonella* effector protein AvrA mediates bacterial intracellular survival during infection by inhibiting MAPK4 and MAPK7 [273]. Bacteria also subvert host immune responses by directly interacting with inhibitory receptors such as the immunoreceptor tyrosine-based inhibitory motif- (ITIM-) bearing inhibitory receptor or through virulence factors that mimic intermediates of host inhibitory signaling [274]. For instance, *S. aureus* targets the ITIM-bearing inhibitory receptor paired Ig-like receptor B (PIR-B) to reduce TLR-induced inflammatory cytokine release by macrophages during infection [275]. *Helicobacter pylori* releases effector proteins, which contain ITIM-like motifs within host cells and suppress immune responses [276]. On the other hand, *Yersinia* and *Salmonella* impair inflammatory signaling by secreting effectors that resemble host cellular protein tyrosine phosphatases [274]. The blockade of these inhibitory receptors may be a novel strategy to improve the host-mediated immunity against persistent pathogens.

#### 3.2.2. Evasion of Autophagy

Autophagy is a process that engulfs and delivers cytoplasmic constituents for lysosomal degradation and is a target for maintaining persistence by intracellular pathogens. *L. monocytogenes* evades autophagic recognition by proteins ActA and internalin K [247] while *L. pneumophila* effector protein RavZ inhibits autophagy through irreversible Atg8 protein deconjugation attached on autophagosome membranes [277]. Some intracellular bacterial pathogens, e.g., *Anaplasma phagocytophilum*, lives within an autophagosome and inhibits autophagosomal-lysosomal fusion by secreting protein Anaplasma translocated substrate 1 that hijacks the Beclin 1-Atg14L autophagy initiation pathway [215]. Viruses are very adept in evading autophagy early during autophagosome formation and during autophagosomal-lysosomal fusion. For example, TRIM proteins were found to regulate autophagy by HSV-1 and influenza A virus *via* the TRIM23-TBK1-p62 axis as a key component of selective autophagy [278]. Picornaviruses including poliovirus and food-and-mouth disease virus subvert autophagy and generate unique replication organelles for their multiplication [279]. Similarly, HCV triggers Golgi fragmentation and autophagy through the immunity-related GTPase M [280]. Evasion of autophagy is also used by RNA viruses that replicate in the nucleus, e.g., HIV, which inhibits autophagosome maturation *via* Tat, Nef, and Vpu proteins [281].

#### 3.2.3. Inhibition of Complement Proteins

The complement system is another target for persistent pathogens aiming at evading the host innate immune response. Viruses like HCMV, HIV, and human lymphoma virus type I incorporate complement inhibitor proteins DAF, MCP, and CD59 in their envelope during virus release from the cell [282] while others like poxvirus and the herpesviruses encode homologues of complement inhibitors. A number of bacteria express surface proteins that can bind C4BP (classical/lectin pathway) or factor H (alternative pathway) and thereby prevent their cofactor functions in factor I-mediated cleavage of C3b/C4b and subsequent complement activation [283]. Among persistent bacterial pathogens, *Neisseria* is a classical example for evading complement activation. *N. gonorrhea* expresses two kinds of porin molecules, Por1A and Por1B, that binds complement component C4BP [284].

#### 3.2.4. Inhibition of Cytokines and Chemokines

Inhibiting the production of cytokines, such as type I and II interferons, TNFs, and IL-1, and chemokines is another way to escape host immune responses, and such strategies have been very well documented for viral infections [285]. In addition, large DNA viruses (herpes and poxviruses) are able to express surface proteins that mimic cytokine and cytokine receptors [286]. Other viruses modulate the chemokine network by producing their own versions of chemokines or chemokine receptors or by secreting chemokine-binding proteins, not found in the host [286]. Persistent bacterial pathogens can manipulate the cytokine network by producing effector proteins, which inhibit cytokine release such as TNF-*α* release in *Yersinia enterocolitica* [287] and *Brucella suis* [288] and IL-2 in *S. typhimurium* [289], while *Legionella pneumophila* degrades IL-2 by producing a Zn metalloproteinase [290].

#### 3.2.5. Inhibition of Adaptive Immune Responses

Adaptive immune responses are critical for the clearance of bacterial and viral infections. However, persistent pathogens have acquired various mechanisms to counteract the adaptive immune response at various levels. In viral infections, NK cells are part of the first line of cellular defense, which can be countered through expression of viral proteins blocking either NK-cell receptor function, cytokine release, or MHC-I homologs [291]. HBV suppresses NK cell function by upregulating the inhibitory molecule, T cell immunoglobulin, and mucin protein-3 (Tim-3) on NK cells [292] while HCV inhibits NK cell activity by crosslinking CD81 with its viral glycoprotein E2 [293]. Viral interference with proteasome cleavage, translocation through the transporters associated with antigen processing, and presentation through MHC class I as well as MHC class II have been documented for persistent infections with HIV, HSV, HPV, HCMV, and adenovirus [285]. Viruses can interfere with DC functions in many ways and modulate their effector functions [294]. Viruses also evade neutralizing antibodies; e.g., cell-to-cell spread of HCV prevents antibody-virion contact [295], and mutations in glycoproteins of both HCV [296] and HPV [297] reduce host antibody reactivity. Among bacterial pathogens, *N. gonorrhea* manipulates host immune responses by inhibition of T lymphocyte activation and proliferation (mediated by the Opa protein) [298]. A vacuolating immunotoxin, VacA, produced by *H. pylori*, inhibits proliferation of T lymphocytes *via* the TCR-IL-2 signaling pathway [299]. Other bacterial pathogens reduce MHC antigen presentation and evade host T cell response; e.g., *M. tuberculosis*-infected cells export antigen for uptake and presentation by uninfected bystander cells, which reduce MHC class II antigen presentation by infected cells and limits host-mediated CD4^+^ T cell control [300]. *B. abortus* infection inhibits the expression of MHC-II molecules by IL-6-dependent inhibition of transactivator (CIITA), which prevents its recognition by T cells establishing a chronic infection [301]. Another evasion strategy adopted by bacterial pathogens is to secrete enzymes such as IgA proteases that degrade immunoglobulins; e.g., secreted IgA protease from *N. meningitidis* is transported to the nucleus of infected cells where it cleaves the p65/RelA component of the NF-*κ*B complex [302]. Immune checkpoint inhibitors, e.g., CTLA-4, programmed death- (PD-) 1, lymphocyte-activation gene 3 (LAG-3), and Tim-3, are today well recognized in the immune evasion of cancers [303]. Microbial pathogens can also exploit immune checkpoint inhibitors to limit host-mediated antigen-specific immune responses; e.g., *S. aureus* modulates PD-ligand 1 to evade immune activation [304]. In *Plasmodium falciparum* infection, Tim-3 on immune cells negatively regulates cell-mediated immunity, the blockade of which improves protection against malaria [305]. Similarly, Tim-3 mediates T cell exhaustion during *M. tuberculosis* infection [306]. PD-1 has been implicated in the regulation of T cell responses during HIV, HCV, and HBV infection [307]. Immune checkpoint blockade may be an important novel strategy for managing chronic infections, which presently lack effective therapies or vaccines [307].

#### 3.2.6. Suppression of Cell Death

Induction of cell death is one of the canonical strategies used by phagocytes to clear intracellular pathogens by expelling microbes from their replicative niche. Successful intracellular pathogens modulate different forms of cell death such as apoptosis, pyroptosis, necrosis/necroptosis, and NETosis, to evade host immune defense [308]. Apoptosis is an active programmed cell death, which does not induce inflammation but is dependent on sequential proteolytic activation of caspases. Cellular proteins involved in the control of apoptosis, such as FLIP, caspase inhibitor, selenoproteins, ligands of the TNF family, Bcl-2, and p53, are targeted by viral antiapoptotic mechanisms such as inhibition of multiple caspases and TNF-induced apoptosis, inactivation of p53, and homologs of Bcl-2 [285]. A number of virus-encoded proteins interfere with caspase activation or inhibit caspase activity and avoid apoptosis of host cells for their survival; e.g., the HSV-1 latency-associated transcript blocks apoptosis and inhibits caspase-3 activation [309]. Bacterial infections may drive the antiapoptotic pathways through production of bacterial toxins as in *Listeria* infection or secretion of effector proteins and T3SS as in *Salmonella* and *Yersinia* infections [310, 311] or by blocking proapoptotic proteins Bax and Bak or activate caspase-3 as in *Chlamydia* infection [312]. However, recently it was reported that although *C. trachomatis*-infected cells are protected from apoptosis at early and mid-stages of infection, they remain susceptible to the induction of other cell death modalities, especially necrosis [313]. It was also shown that this necrotic death occurred with similar kinetics as apoptosis in uninfected cells, which indicates that *C. trachomatis* fails to significantly prolong the lifespan of its host cell when exposed to proapoptotic insults [313]. *Rickettsia rickettsii* inhibits apoptosis through induction of NF-*κ*B-mediated events, and as a result, the infected host cell remains at the site of infection [314]. *Coxiella burnetii* effector protein CaeA interferes with the intrinsic and extrinsic apoptosis pathway [315]. Necrosis is a caspase-independent pathological cell death, which triggers inflammation and results in extensive tissue damage [308]. *M. tuberculosis* infects macrophages and induces necrosis to avoid immune response and to disseminate [316]. Necroptosis is a form of regulated necrosis that depends on activation of the necrosome, which is a protein complex in which receptor-interacting protein kinase 3 (RIPK3) is activated. Vaccinia, influenza, and HSV-1 are among many viruses that induce necroptosis *via* their effector proteins binding to RIPK3 [317]. Pyroptosis is a highly inflammatory form of programmed cell death mediated by gasdermin and requires the caspase-1 activation in inflammasomes. Various studies have demonstrated pyroptotic death of macrophages and dendritic cells infected with intracellular pathogens as one of the key mechanisms for host survival [318].

## 4. Conclusion

During infections, there is a constant combat between pathogens that attempt to establish and maintain an infection and host immune defense mechanisms to prevent such establishment. The outcome of this battle is determined by many factors related to host, pathogen, and the immune responses. In this review, we highlight host immune defense mechanisms against microbial infections and the various anti-immune strategies adopted by the intracellular pathogens to thwart this immune defense and establish persistent infections. New technological advancements in the field of immunology such as genomics, proteomics, RNA sequencing, and imaging have allowed track of intracellular persistent infections and the associated cellular changes. Combining all these robust immunological techniques with animal models of infectious diseases, including transgenic and humanized animal models, provides detailed information of chemical, epigenetic, and cellular interactions that occur during persistent infections. Although recent progress has brought us closer to understanding the mechanisms of pathogen persistence and counteractive host immunity, a lot more is still to be explored to completely translate the host-pathogen interactions during persistent intracellular infections. An interdisciplinary approach will be critical to bridging the knowledge gaps in infection dynamics during persistent infections. With the global presence of emerging and reemerging infectious diseases and classical infections continuously present, an improved understanding of this knowledge is crucial for developing improved disease diagnostics, interventional strategies, or novel vaccines.

## Figures and Tables

**Table 1:** Lymphocyte subsets in the control of microbial infections.

Lymphocyte subset	Antigen presentation	Transcription factors	Effector molecules secreted	Mechanism	Evidence for control in intracellular infections (gene deficiency or direct involvement)
Th1	MHC class II	T-bet, STAT4, STAT1	IFN-*γ*, TNF-*α*, IL-2, lymphotoxin *α*	Activation of macrophages by IFN-*γ*, upregulation of iNOS and ROI, proliferation of CTL	IFN-*γ* -/- [16–21]
					TNF-*α* -/- [22–29]
					IL-12p40 -/- [30–32]
					IL-18 -/- [33–35]

Th2	MHC class II	GATA3, STAT5, STAT6	IL-4, IL-5, IL-9, IL-13	Stimulate B cells, antibody production, antibody class switching	Th2 cytokines [30–32]

Th17	MHC class II	ROR*γ*t, STAT3	IL-17A, IL-17F, IL-21, IL-22, CCL20	Recruitment, activation and migration of neutrophils	IL-17 -/- [36–41]
					IL-17 RA -/- [42–47]
					IFN-*γ* -/- [16, 18, 19, 48]
					IL-23 -/- [31, 49–51]

Tfh	MHC class II	Bcl6, c-MAF	IL-10, IL-21	Provides help for B cells to allow formation of plasma cells and memory B cells	Tfh -/- [52, 53]
					IL-21 -/- [54]
					IL-6 -/- [55]

Tregs	MHC class II	FOXP3, SMAD, STAT5	IL-10, TGF-*β*, IL-35	Immunosuppression and tolerance	CD4^+^ Tregs [56–58]
					CD8^+^ Tregs [59–61]
					IL-10 -/- [57, 62, 63]
					TGF-*β* -/- [64, 65]

CD8^+^/CTL	MHC class I	EOMES, BLIMP1	IFN-*γ*, perforin, granzyme, granulysin, FAS-FAS ligand	Cytotoxicity, programmed cell death by caspase or receptor-mediated FAS-FAS ligand apoptosis	IFN-*γ* -/- [66–71]
					TNF-*α* -/- [22–28, 72]
					Perforin -/- [73–75]
					Granzyme -/- [75, 76]

*γδ* T	CD1c	PLZF, GATA3, TBX21	IFN-*γ*, IL-17A, IL-17F, IL-22	Pro- and anti-inflammatory functions at epithelial surfaces	*γδ* TCR -/- [77–82]
					IL-17 [37, 38, 46, 83]
					IL-22 [84]

NK	MHC class I are inhibitory	PU.1, Ets-1, GATA3, IRF-2	IFN-*γ*, TNF-*α*, perforin, granzyme, *α*-defensins	Cytotoxic, direct cytolysis by apoptosis, ADCC	NK -/- [85, 86]
					IFN-*γ* -/- [87, 88]
					Perforin -/- [87, 89]

iNKT	CD1d	PLZF, TBX21, ERK	IL-4, IFN-*γ*, IL-17A, GM-CSF	Pro- and anti-inflammatory functions	iNKT cells [90–95]

MAIT	MR1	ZBTB16, ROR(*γ*t)	IFN-*γ*, TNF-*α*, IL-17, granzyme	Cytokine production, cytotoxic	MAIT -/- [96–98]
					MR -/- [99–101]

B	NA	PU.1, Pax5 Ikaros	Immunoglobulins, IL-10	Antibody secretion, neutralization, opsonization, phagocytosis, antigen presentation	B cells [102–108]
					Polymeric-Ig receptor -/- [109–113]

ADCC: antibody-dependent cellular cytotoxicity; B: B lymphocyte; Bcl6: B cell lymphoma 6; BLIMP1: PR domain zinc finger protein 1; CCL: chemokine ligand; CD: cluster of differentiation; c-MAF: c-musculoaponeurotic fibrosarcoma oncogene homolog; CTL: cytotoxic T lymphocyte; EOMES: Eomesodermin; ERK: extracellular signal-regulated kinase; Ets-1: erythroblastosis virus transcription factor-1; FOXP3: Forkhead box P3; GATA, trans-acting T cell-specific transcription factor; *γδ* T: gamma delta T cells; GM-CSF: granulocyte-macrophage colony-stimulating factor; IFN-*γ*: interferon gamma; Ig: immunoglobulin; IL: interleukin; IL-17RA: interleukin 17 receptor a; iNKT: invariant natural killer T cell; iNOS: inducible nitric oxide synthase; IRF-2: interferon regulatory factor 2; MHC: major histocompatibility complex; MR1: major histocompatibility complex class I-related gene protein; MAIT: mucosal-associated invariant T cells; NA: not applicable; NK: natural killer cells; Pax5: paired box protein 5; PLZF: promyelocytic leukemia zinc finger; ROR*γ*t: RAR-related orphan receptor gamma 2; ROI: reactive oxygen intermediates; STAT: signal transducer and activator of transcription; TBX: T-box transcription factor; Tfh: follicular helper T cells; TGF-*β*: transforming growth factor beta; Th: helper T cells; TNF-*α*: tumor necrosis factor alpha; Tregs: regulatory T cells; ZBTB16: zinc finger and BTB domain-containing protein 16.

**Table 2:** Selected mechanisms for evasion of host defense by persistent intracellular pathogens.

Mechanism	Pathogen(s)	Pathogen type	Remarks	Reference(s)
Immunomodulation	*Salmonella* spp.	B	Lipid A modification	[186]
	*Leptospira interrogans*	B	Peptidoglycan modification	[187, 188]
	Poxvirus	V	Host cytokine decoy receptors	[189]
	Herpesvirus	V	Host cytokine and chemokine decoy receptors	[190]
	*Mycobacterium tuberculosis*	B	ESX secretion system	[191]
	*Salmonella typhimurium*	B	Type III secretion system	[192]
	*Brucella abortus*	B	Type IV secretion system	[193]
	*Staphylococcus aureus*	B	Ess secretion system	[194]
	*Bacillus subtilis*	B	Yuk/Yue secretion system	[195]

Antigenic variation	Influenza virus	V	Antigenic drift/shift	[196]
	*Neisseria* spp.	B	DNA rearrangement	[197, 198]
	*Plasmodium* spp.	P	Programmed gene rearrangement	[199]
	*S. Typhimurium*	B	DNA rearrangement	[200]
	*Trypanosoma brucei*	P	Programmed gene rearrangement	[201]
	Hepatitis C virus	V	DNA rearrangement	[202]
	Human immunodeficiency virus	V	DNA rearrangement	[203]

Hiding in safe target cells/tissues	Epstein-Barr virus	V	B cells	[204]
	Herpes simplex virus	V	Sensory neurons	[27, 205]
	*Leishmania* spp.	P	Fibroblasts	[206]
	*Mycobacterium leprae*	B	Peripheral nerves (Schwann cells)	[207]
	*Salmonella enterica* Typhi	B	Reticuloendothelial system	[208]
	*Toxoplasma* spp.	P	Cerebellar neurons	[209]
	Varicella zoster virus	V	Dorsal root ganglia	[210]

B: bacteria; P: protozoa; V: virus.

**Table 3:** Selected mechanisms for modulation/suppression of host immune response by persistent intracellular pathogens.

Mechanism	Pathogen(s)	Pathogen type	Remarks	Reference(s)
Subversion of host defense	*Brucella* spp.	B	Inhibit fusion with host lysosomal compartment and alter lysosomal pH	[211]
	*Chlamydiae* spp.	B	Degradation of host proteins and deactivation of neutrophils by chlamydial protease-like activating factor	[212, 213]
	*Francisella tularensis*	B	Escape into cytosol	[214]
	*Anaplasma phagocytophilum*	B	Inhibits autophagosomal-lysosomal fusion	[215]
	*Legionella pneumophila*	B	Membrane-bound vacuole and effector protein (Ank protein) release	[216]
	*Listeria monocytogenes*	B	Escape into cytosol	[217]
	*Mycobacterium tuberculosis*	B	Inhibition of phagolysosome formation	[183]
	*Rickettsia* spp.	B	Escape into cytosol and replicate in cytoplasm of host cell	[218]
	*Salmonella enterica* Typhi	B	Inhibit fusion with host lysosomal compartment and alter lysosomal pH	[36, 219, 220]
	*Toxoplasma gondii*	B	Generate own vesicle	[221]

Resistance to host effector mechanisms	Cytomegalovirus	V	Inhibition of humoral immunity and inflammatory response. Blockage of Ag processing and presentation	[63]
	Epstein-Barr virus	V	Inhibition of inflammatory response	[204, 222]
	Herpes simplex virus	V	Inhibition of humoral immunity and blockage of Ag processing and presentation	[27, 205]
	*Leishmania* spp.	P	Silent phagocytosis	[206]
	*Mycobacterium tuberculosis*	B	Ability to persist in macrophages	[183]
	Vaccinia virus	V	Inhibition of humoral immunity and inflammatory response	[223–225]

Induction of inappropriate immune responses/immunosuppression/Tregs	Hepatitis C virus (HCV)	B	Immunosuppression by complement regulatory pathway	[160, 226–228]
	*Mycobacterium leprae*	B	Immunosuppression of Th2 cytokines, indoleamine 2, 3-dioxygenase	[159, 229]
	HCV	V	Induction of Tregs	[160, 227]
	Human immunodeficiency virus	V	Induction of Tregs	[161]
	*Leishmania major*	P	Induction of Tregs	[103, 230]
	*M. Tuberculosis*	B	Induction of Tregs	[231]
	*Plasmodium* spp.	P	Induction of Tregs	[232]

Ag: antigen; B: bacteria; P: protozoa; Th2: type 2 helper T cells; Tregs: regulatory T cells; V: virus.

## References

[1] Grant S. S., Hung D. T. (2013). Persistent bacterial infections, antibiotic tolerance, and the oxidative stress response. *Virulence*.

[2] Belkaid Y., Hand T. W. (2014). Role of the microbiota in immunity and inflammation. *Cell*.

[3] Price L. B., Hungate B. A., Koch B. J., Davis G. S., Liu C. M. (2017). Colonizing opportunistic pathogens (COPs): the beasts in all of us. *PLoS Pathogens*.

[4] Bellini W. J., Rota J. S., Lowe L. E. (2005). Subacute sclerosing panencephalitis: more cases of this fatal disease are prevented by measles immunization than was previously recognized. *The Journal of Infectious Diseases*.

[5] Kaufmann S. H. E. (2011). Intracellular pathogens: living in an extreme environment. *Immunological Reviews*.

[6] Cossart P., Helenius A. (2014). Endocytosis of viruses and bacteria. *Cold Spring Harbor Perspectives in Biology*.

[7] Foley J. (2015). Mini-review: strategies for variation and evolution of bacterial antigens. *Computational and Structural Biotechnology Journal*.

[8] Iwasaki A., Medzhitov R. (2015). Control of adaptive immunity by the innate immune system. *Nature Immunology*.

[9] Schenten D., Medzhitov R. (2011). The control of adaptive immune responses by the innate immune system. *Advances in Immunology*.

[10] Netea M. G., Joosten L. A. B., Latz E. (2016). Trained immunity: a program of innate immune memory in health and disease. *Science*.

[11] Netea M. G., Quintin J., van der Meer J. W. M. (2011). Trained immunity: a memory for innate host defense. *Cell Host & Microbe*.

[12] Lerm M., Netea M. G. (2016). Trained immunity: a new avenue for tuberculosis vaccine development. *Journal of Internal Medicine*.

[13] Sanchez-Ramon S., Conejero L., Netea M. G., Sancho D., Palomares O., Subiza J. L. (2018). Trained immunity-based vaccines: a new paradigm for the development of broad-spectrum anti-infectious formulations. *Frontiers in Immunology*.

[14] Arts R. J. W., Joosten L. A. B., Netea M. G. (2018). The potential role of trained immunity in autoimmune and autoinflammatory disorders. *Frontiers in Immunology*.

[15] Zhu Y., Yao S., Chen L. (2011). Cell surface signaling molecules in the control of immune responses: a tide model. *Immunity*.

[16] Cooper A. M., Dalton D. K., Stewart T. A., Griffin J. P., Russell D. G., Orme I. M. (1993). Disseminated tuberculosis in interferon gamma gene-disrupted mice. *Journal of Experimental Medicine*.

[17] Jupelli M., Selby D. M., Guentzel M. N. (2010). The contribution of interleukin-12/interferon-*γ* axis in protection against neonatal pulmonary *Chlamydia muridarum* challenge. *Journal of Interferon & Cytokine Research*.

[18] McCall M. B. B., Sauerwein R. W. (2010). Interferon-*γ*—central mediator of protective immune responses against the pre-erythrocytic and blood stage of malaria. *Journal of Leukocyte Biology*.

[19] Elkins K. L., Colombini S. M., Meierovics A. I., Chu M. C., Chou A. Y., Cowley S. C. (2010). Survival of secondary lethal systemic *Francisella* LVS challenge depends largely on interferon gamma. *Microbes and Infection*.

[20] Kima P. E., Soong L. (2013). Interferon gamma in leishmaniasis. *Frontiers in Immunology*.

[21] Walker D. H., Dumler J. S. (2015). The role of CD8 T lymphocytes in rickettsial infections. *Seminars in Immunopathology*.

[22] Dharmana E., Keuter M., Netea M. G., Verschueren I. C., Kullberg B. J. (2002). Divergent effects of tumor necrosis factor-alpha and lymphotoxin-alpha on lethal endotoxemia and infection with live *Salmonella typhimurium* in mice. *European Cytokine Network*.

[23] Murthy A. K., Li W., Chaganty B. K. R. (2011). Tumor necrosis factor alpha production from CD8^+^ T cells mediates oviduct pathological sequelae following primary genital *Chlamydia muridarum* infection. *Infection and Immunity*.

[24] Macedo G. C., Magnani D. M., Carvalho N. B., Bruna-Romero O., Gazzinelli R. T., Oliveira S. C. (2008). Central role of MyD88-dependent dendritic cell maturation and proinflammatory cytokine production to control *Brucella abortus* infection. *The Journal of Immunology*.

[25] Sonje M. B., Abram M., Stenzel W., Deckert M. (2010). *Listeria monocytogenes* (delta-*act*A mutant) infection in tumor necrosis factor receptor p55-deficient neonatal mice. *Microbial Pathogenesis*.

[26] Cowley S. C., Elkins K. L. (2011). Immunity to *francisella*. *Frontiers in Microbiology*.

[27] Sergerie Y., Rivest S., Boivin G. (2007). Tumor necrosis factor-*α* and interleukin-1*β* play a critical role in the resistance against lethal herpes simplex virus encephalitis. *The Journal of Infectious Diseases*.

[28] Miura Y., Misawa N., Kawano Y. (2003). Tumor necrosis factor-related apoptosis-inducing ligand induces neuronal death in a murine model of HIV central nervous system infection. *Proceedings of the National Academy of Sciences of the United States of America*.

[29] Allie N., Grivennikov S. I., Keeton R. (2013). Prominent role for T cell-derived tumour necrosis factor for sustained control of *Mycobacterium tuberculosis* infection. *Scientific Reports*.

[30] Martínez-Barricarte R., Markle J. G., Ma C. S. (2018). Human IFN-*γ* immunity to mycobacteria is governed by both IL-12 and IL-23. *Science Immunology*.

[31] Awoniyi M., Miller S. I., Wilson C. B., Hajjar A. M., Smith K. D. (2012). Homeostatic regulation of *Salmonella*-induced mucosal inflammation and injury by IL-23. *PLoS One*.

[32] Henry C. J., Grayson J. M., Brzoza-Lewis K. L. (2010). The roles of IL-12 and IL-23 in CD8^+^ T cell-mediated immunity against *Listeria monocytogenes*: insights from a DC vaccination model. *Cellular Immunology*.

[33] Schneider B. E., Korbel D., Hagens K. (2010). A role for IL-18 in protective immunity against *Mycobacterium tuberculosis*. *European Journal of Immunology*.

[34] Oliveira A. C., Gomes-Neto J. F., Barbosa C. H. D. (2017). Crucial role for T cell-intrinsic IL-18R-MyD88 signaling in cognate immune response to intracellular parasite infection. *eLife*.

[35] Pham O. H., O’Donnell H., al-Shamkhani A., Kerrinnes T., Tsolis R. M., McSorley S. J. (2017). T cell expression of IL-18R and DR3 is essential for non-cognate stimulation of Th1 cells and optimal clearance of intracellular bacteria. *PLoS Pathogens*.

[36] Schulz S. M., Kohler G., Schutze N. (2008). Protective immunity to systemic infection with attenuated *Salmonella enterica* serovar *enteritidis* in the absence of IL-12 is associated with IL-23-dependent IL-22, but not IL-17. *The Journal of Immunology*.

[37] Khader S. A., Pearl J. E., Sakamoto K. (2005). IL-23 compensates for the absence of IL-12p70 and is essential for the IL-17 response during tuberculosis but is dispensable for protection and antigen-specific IFN-*γ* responses if IL-12p70 is available. *The Journal of Immunology*.

[38] Umemura M., Yahagi A., Hamada S. (2007). IL-17-mediated regulation of innate and acquired immune response against pulmonary *Mycobacterium bovis* bacille Calmette-Guerin infection. *The Journal of Immunology*.

[39] Bai H., Cheng J., Gao X. (2009). IL-17/Th17 promotes type 1 T cell immunity against pulmonary intracellular bacterial infection through modulating dendritic cell function. *The Journal of Immunology*.

[40] Wozniak T. M., Saunders B. M., Ryan A. A., Britton W. J. (2010). *Mycobacterium bovis* BCG-specific Th17 cells confer partial protection against *Mycobacterium tuberculosis* infection in the absence of gamma interferon. *Infection and Immunity*.

[41] Roberts L. M., Davies J. S., Sempowski G. D., Frelinger J. A. (2014). IFN-*γ*, but not IL-17A, is required for survival during secondary pulmonary *Francisella tularensis* live vaccine stain infection. *Vaccine*.

[42] Feinen B., Jerse A. E., Gaffen S. L., Russell M. W. (2010). Critical role of Th17 responses in a murine model of *Neisseria gonorrhoeae* genital infection. *Mucosal Immunology*.

[43] Wang X., Chan C. C. S., Yang M. (2011). A critical role of IL-17 in modulating the B-cell response during H5N1 influenza virus infection. *Cellular & Molecular Immunology*.

[44] Kelly M. N., Kolls J. K., Happel K. (2005). Interleukin-17/interleukin-17 receptor-mediated signaling is important for generation of an optimal polymorphonuclear response against *Toxoplasma gondii* infection. *Infection and Immunity*.

[45] Boari J. T., Vesely M. C. A., Bermejo D. A. (2012). IL-17RA signaling reduces inflammation and mortality during *Trypanosoma cruzi* infection by recruiting suppressive IL-10-producing neutrophils. *PLoS Pathogens*.

[46] Okamoto Yoshida Y., Umemura M., Yahagi A. (2010). Essential role of IL-17A in the formation of a mycobacterial infection-induced granuloma in the lung. *The Journal of Immunology*.

[47] Findlay E. G., Greig R., Stumhofer J. S. (2010). Essential role for IL-27 receptor signaling in prevention of Th1-mediated immunopathology during malaria infection. *The Journal of Immunology*.

[48] Rosas L. E., Keiser T., Pyles R., Durbin J., Satoskar A. R. (2003). Development of protective immunity against cutaneous leishmaniasis is dependent on STAT1-mediated IFN signaling pathway. *European Journal of Immunology*.

[49] Khader S. A., Guglani L., Rangel-Moreno J. (2011). IL-23 is required for long-term control of *Mycobacterium tuberculosis* and B cell follicle formation in the infected lung. *The Journal of Immunology*.

[50] Meeks K. D., Sieve A. N., Kolls J. K., Ghilardi N., Berg R. E. (2009). IL-23 is required for protection against systemic infection with *Listeria monocytogenes*. *The Journal of Immunology*.

[51] Markel G., Bar-Haim E., Zahavy E. (2010). The involvement of IL-17A in the murine response to sub-lethal inhalational infection with *Francisella tularensis*. *PLoS One*.

[52] Cook K. D., Shpargel K. B., Starmer J. (2015). T follicular helper cell-dependent clearance of a persistent virus infection requires T cell expression of the histone demethylase UTX. *Immunity*.

[53] Wang X., Dong Q., Li Q. (2018). Dysregulated response of follicular helper T cells to hepatitis B surface antigen promotes HBV persistence in mice and associates with outcomes of patients. *Gastroenterology*.

[54] Moretto M. M., Hwang S., Khan I. A. (2017). Downregulated IL-21 response and T follicular helper cell exhaustion correlate with compromised CD8 T cell immunity during chronic toxoplasmosis. *Frontiers in Immunology*.

[55] Eto D., Lao C., DiToro D. (2011). IL-21 and IL-6 are critical for different aspects of B cell immunity and redundantly induce optimal follicular helper CD4 T cell (Tfh) differentiation. *PLoS One*.

[56] Fan R., Xiang Y., Yang L. (2016). Impaired NK cells’ activity and increased numbers of CD4 + CD25+ regulatory T cells in multidrug-resistant *Mycobacterium tuberculosis* patients. *Tuberculosis*.

[57] Bunn P. T., Montes de Oca M., de Labastida Rivera F. (2018). Distinct roles for CD4^+^ Foxp3^+^ regulatory T cells and IL-10–mediated immunoregulatory mechanisms during experimental visceral Leishmaniasis caused by *Leishmania donovani*. *The Journal of Immunology*.

[58] Kurup S. P., Obeng-Adjei N., Anthony S. M. (2017). Regulatory T cells impede acute and long-term immunity to blood-stage malaria through CTLA-4. *Nature Medicine*.

[59] Joosten S. A., van Meijgaarden K. E., van Weeren P. C. (2010). *Mycobacterium tuberculosis* peptides presented by HLA-E molecules are targets for human CD8 T-cells with cytotoxic as well as regulatory activity. *PLoS Pathogens*.

[60] Li S., Vriend L. E. M., Nasser I. A. (2012). Hepatitis C virus-specific T-cell-derived transforming growth factor beta is associated with slow hepatic fibrogenesis. *Hepatology*.

[61] Fogg M., Murphy J. R., Lorch J., Posner M., Wang F. (2013). Therapeutic targeting of regulatory T cells enhances tumor-specific CD8+ T cell responses in Epstein–Barr virus associated nasopharyngeal carcinoma. *Virology*.

[62] Redford P. S., Boonstra A., Read S. (2010). Enhanced protection to *Mycobacterium tuberculosis* infection in IL-10-deficient mice is accompanied by early and enhanced Th1 responses in the lung. *European Journal of Immunology*.

[63] Tang-Feldman Y. J., Lochhead G. R., Lochhead S. R., Yu C., Pomeroy C. (2011). Interleukin-10 repletion suppresses pro-inflammatory cytokines and decreases liver pathology without altering viral replication in murine cytomegalovirus (MCMV)-infected IL-10 knockout mice. *Inflammation Research*.

[64] Windish H. P., Lin P. L., Mattila J. T. (2009). Aberrant TGF-*β* signaling reduces T regulatory cells in ICAM-1-deficient mice, increasing the inflammatory response to *Mycobacterium tuberculosis*. *Journal of Leukocyte Biology*.

[65] Li C., Sanni L. A., Omer F., Riley E., Langhorne J. (2003). Pathology of *Plasmodium chabaudi chabaudi* infection and mortality in interleukin-10-deficient mice are ameliorated by anti-tumor necrosis factor alpha and exacerbated by anti-transforming growth factor *β* antibodies. *Infection and Immunity*.

[66] Walker D. H., Olano J. P., Feng H. M. (2001). Critical role of cytotoxic T lymphocytes in immune clearance of rickettsial infection. *Infection and Immunity*.

[67] Brandão A. P. M. S., Oliveira F. S., Carvalho N. B. (2012). Host susceptibility to *Brucella abortus* infection is more pronounced in IFN-*γ* knockout than IL-12/*β*2-microglobulin double-deficient mice. *Clinical and Developmental Immunology*.

[68] Gideon H. P., Phuah J., Myers A. J. (2015). Variability in tuberculosis granuloma T cell responses exists, but a balance of pro- and anti-inflammatory cytokines is associated with sterilization. *PLoS Pathogens*.

[69] Nogueira C. V., Zhang X., Giovannone N., Sennott E. L., Starnbach M. N. (2015). Protective immunity against *Chlamydia trachomatis* can engage both CD4^+^ and CD8^+^ T cells and bridge the respiratory and genital mucosae. *The Journal of Immunology*.

[70] Martin M. D., Badovinac V. P. (2015). Antigen-dependent and -independent contributions to primary memory CD8 T cell activation and protection following infection. *Scientific Reports*.

[71] Roberts L. M., Powell D. A., Frelinger J. A. (2018). Adaptive immunity to *Francisella tularensis* and considerations for vaccine development. *Frontiers in Cellular and Infection Microbiology*.

[72] Chakravarty S. D., Zhu G., Tsai M. C. (2008). Tumor necrosis factor blockade in chronic murine tuberculosis enhances granulomatous inflammation and disorganizes granulomas in the lungs. *Infection and Immunity*.

[73] Schmidt N. W., Khanolkar A., Hancox L., Heusel J. W., Harty J. T. (2012). Perforin plays an unexpected role in regulating T-cell contraction during prolonged *Listeria monocytogenes* infection. *European Journal of Immunology*.

[74] de Alencar B. C. G., Persechini P. M., Haolla F. A. (2009). Perforin and gamma interferon expression are required for CD4^+^ and CD8^+^ T-cell-dependent protective immunity against a human parasite, *Trypanosoma cruzi*, elicited by heterologous plasmid DNA prime-recombinant adenovirus 5 boost vaccination. *Infection and Immunity*.

[75] Sanapala S., Yu J. J., Murthy A. K. (2012). Perforin- and granzyme-mediated cytotoxic effector functions are essential for protection against *Francisella tularensis* following vaccination by the defined *F. tularensis* subsp. *novicida* Δ*fopC* vaccine strain. *Infection and Immunity*.

[76] Tang H., Li C., Wang L., Zhang H., Fan Z. (2012). Granzyme H of cytotoxic lymphocytes is required for clearance of the hepatitis B virus through cleavage of the hepatitis B virus X protein. *The Journal of Immunology*.

[77] Simonian P. L., Roark C. L., Wehrmann F. (2009). IL-17A-expressing T cells are essential for bacterial clearance in a murine model of hypersensitivity pneumonitis. *The Journal of Immunology*.

[78] Skyberg J. A., Thornburg T., Rollins M., Huarte E., Jutila M. A., Pascual D. W. (2011). Murine and bovine *γδ* T cells enhance innate immunity against *Brucella abortus* infections. *PLoS One*.

[79] Rhodes K. A., Andrew E. M., Newton D. J., Tramonti D., Carding S. R. (2008). A subset of IL-10-producing gammadelta T cells protect the liver from *Listeria*-elicited, CD8^+^ T cell-mediated injury. *European Journal of Immunology*.

[80] Li B., Bassiri H., Rossman M. D. (1998). Involvement of the Fas/Fas ligand pathway in activation-induced cell death of mycobacteria-reactive human *γδ* T cells: a mechanism for the loss of *γδ* T cells in patients with pulmonary tuberculosis. *The Journal of Immunology*.

[81] Wallace M., Scharko A. M., Pauza C. D. (1997). Functional *γδ* T-lymphocyte defect associated with human immunodeficiency virus infections. *Molecular Medicine*.

[82] Xu S., Han Y., Xu X., Bao Y., Zhang M., Cao X. (2010). IL-17A-producing *γδ*T cells promote CTL responses against *Listeria monocytogenes* infection by enhancing dendritic cell cross-presentation. *The Journal of Immunology*.

[83] Schulz S. M., Kohler G., Holscher C., Iwakura Y., Alber G. (2008). IL-17A is produced by Th17, *γδ* T cells and other CD4^−^ lymphocytes during infection with *Salmonella enterica* serovar *Enteritidis* and has a mild effect in bacterial clearance. *International Immunology*.

[84] Wilson M. S., Feng C. G., Barber D. L. (2010). Redundant and pathogenic roles for IL-22 in mycobacterial, protozoan, and helminth infections. *The Journal of Immunology*.

[85] Zulu M. Z., Naidoo K. K., Mncube Z. (2017). Reduced expression of siglec-7, NKG2A, and CD57 on terminally differentiated CD56^−^CD16^+^ natural killer cell subset is associated with natural killer cell dysfunction in chronic HIV-1 clade C infection. *AIDS Research and Human Retroviruses*.

[86] Varchetta S., Mele D., Lombardi A. (2016). Lack of Siglec-7 expression identifies a dysfunctional natural killer cell subset associated with liver inflammation and fibrosis in chronic HCV infection. *Gut*.

[87] Fang R., Ismail N., Walker D. H. (2012). Contribution of NK cells to the innate phase of host protection against an intracellular bacterium targeting systemic endothelium. *The American Journal of Pathology*.

[88] Goldszmid R. S., Caspar P., Rivollier A. (2012). NK cell-derived interferon-*γ* orchestrates cellular dynamics and the differentiation of monocytes into dendritic cells at the site of infection. *Immunity*.

[89] Müller A. A., Dolowschiak T., Sellin M. E. (2016). An NK cell perforin response elicited via IL-18 controls mucosal inflammation kinetics during *Salmonella* gut infection. *PLoS Pathogens*.

[90] Mureithi M. W., Cohen K., Moodley R. (2011). Impairment of CD1d-restricted natural killer T cells in chronic HIV type 1 clade C infection. *AIDS Research and Human Retroviruses*.

[91] Paget C., Ivanov S., Fontaine J. (2012). Interleukin-22 is produced by invariant natural killer T lymphocytes during influenza A virus infection: potential role in protection against lung epithelial damage. *Journal of Biological Chemistry*.

[92] Jiang X., Zhang M., Lai Q. (2011). Restored circulating invariant NKT cells are associated with viral control in patients with chronic hepatitis B. *PLoS One*.

[93] Renneson J., Guabiraba R., Maillet I. (2011). A detrimental role for invariant natural killer T cells in the pathogenesis of experimental dengue virus infection. *The American Journal of Pathology*.

[94] Joyee A. G., Qiu H., Wang S., Fan Y., Bilenki L., Yang X. (2007). Distinct NKT cell subsets are induced by different *Chlamydia* species leading to differential adaptive immunity and host resistance to the infections. *The Journal of Immunology*.

[95] Chancellor A., White A., Tocheva A. S. (2017). Quantitative and qualitative iNKT repertoire associations with disease susceptibility and outcome in macaque tuberculosis infection. *Tuberculosis*.

[96] Bourhis L. L., Péguillet I., Guihot A. (2010). Antimicrobial activity of mucosal-associated invariant T cells. *Nature Immunology*.

[97] Wilgenburg B., Loh L., Chen Z. (2018). MAIT cells contribute to protection against lethal influenza infection in vivo. *Nature Communications*.

[98] Meierovics A., Yankelevich W.-J. C., Cowley S. C. (2013). MAIT cells are critical for optimal mucosal immune responses during in vivo pulmonary bacterial infection. *Proceedings of the National Academy of Sciences of the United States of America*.

[99] Wang H., D’Souza C., Lim X. Y. (2018). MAIT cells protect against pulmonary *Legionella longbeachae* infection. *Nature Communications*.

[100] D’Souza C., Pediongco T., Wang H. (2018). Mucosal-associated invariant T cells augment immunopathology and gastritis in chronic *Helicobacter pylori* infection. *The Journal of Immunology*.

[101] Chen Z., Wang H., D'Souza C. (2017). Mucosal-associated invariant T-cell activation and accumulation after *in vivo* infection depends on microbial riboflavin synthesis and co-stimulatory signals. *Mucosal Immunology*.

[102] Moore T., Ekworomadu C. O., Eko F. O. (2003). Fc receptor–mediated antibody regulation of T cell immunity against intracellular pathogens. *The Journal of Infectious Diseases*.

[103] Woelbing F., Kostka S. L., Moelle K. (2006). Uptake of *Leishmania major* by dendritic cells is mediated by Fc*γ* receptors and facilitates acquisition of protective immunity. *Journal of Experimental Medicine*.

[104] Joller N., Weber S. S., Muller A. J. (2010). Antibodies protect against intracellular bacteria by Fc receptor-mediated lysosomal targeting. *Proceedings of the National Academy of Sciences of the United States of America*.

[105] Vouldoukis I., Mazier D., Moynet D., Thiolat D., Malvy D., Mossalayi M. D. (2011). IgE mediates killing of intracellular *Toxoplasma gondii* by human macrophages through CD23-dependent, interleukin-10 sensitive pathway. *PLoS One*.

[106] Lu L. L., Chung A. W., Rosebrock T. R. (2016). A functional role for antibodies in tuberculosis. *Cell*.

[107] Bitsaktsis C., Babadjanova Z., Gosselin E. J. (2015). *In vivo* mechanisms involved in enhanced protection utilizing an Fc receptor-targeted mucosal vaccine platform in a bacterial vaccine and challenge model. *Infection and Immunity*.

[108] Mollo S. B., Zajac A. J., Harrington L. E. (2013). Temporal requirements for B cells in the establishment of CD4 T cell memory. *The Journal of Immunology*.

[109] Schwartz-Cornil I., Benureau Y., Greenberg H., Hendrickson B. A., Cohen J. (2002). Heterologous protection induced by the inner capsid proteins of rotavirus requires transcytosis of mucosal immunoglobulins. *Journal of Virology*.

[110] Wijburg O. L. C., Uren T. K., Simpfendorfer K., Johansen F. E., Brandtzaeg P., Strugnell R. A. (2006). Innate secretory antibodies protect against natural *Salmonella typhimurium* infection. *Journal of Experimental Medicine*.

[111] Cunningham K. A., Carey A. J., Finnie J. M. (2008). Poly-immunoglobulin receptor-mediated transport of IgA into the male genital tract is important for clearance of *Chlamydia muridarum* infection. *American Journal of Reproductive Immunology*.

[112] Sahi-Ozaki Y., Yoshikawa T., Iwakura Y. (2004). Secretory IgA antibodies provide cross-protection against infection with different strains of influenza B virus. *Journal of Medical Virology*.

[113] Tjärnlund A., Rodríguez A., Cardona P. J. (2006). Polymeric IgR knockout mice are more susceptible to mycobacterial infections in the respiratory tract than wild-type mice. *International Immunology*.

[114] Levin R., Grinstein S., Canton J. (2016). The life cycle of phagosomes: formation, maturation, and resolution. *Immunological Reviews*.

[115] Flannagan R. S., Jaumouille V., Grinstein S. (2012). The cell biology of phagocytosis. *Annual Review of Pathology: Mechanisms of Disease*.

[116] Berlutti F., Pantanella F., Natalizi T. (2011). Antiviral properties of lactoferrin—a natural immunity molecule. *Molecules*.

[117] Wessling-Resnick M. (2015). Nramp1 and other transporters involved in metal withholding during infection. *Journal of Biological Chemistry*.

[118] Becskei A., Grusby M. J. (2007). Contribution of IL-12R mediated feedback loop to Th1 cell differentiation. *FEBS Letters*.

[119] Snapper C. M., Paul W. E. (1987). Interferon-gamma and B cell stimulatory factor-1 reciprocally regulate Ig isotype production. *Science*.

[120] Schroder K., Hertzog P. J., Ravasi T., Hume D. A. (2004). Interferon-*γ*: an overview of signals, mechanisms and functions. *Journal of Leukocyte Biology*.

[121] Ní Cheallaigh C., Keane J., Lavelle E. C., Hope J. C., Harris J. (2011). Autophagy in the immune response to tuberculosis: clinical perspectives. *Clinical & Experimental Immunology*.

[122] Ohshima J., Lee Y., Sasai M. (2014). Role of mouse and human autophagy proteins in IFN-*γ*-induced cell-autonomous responses against *Toxoplasma gondii*. *The Journal of Immunology*.

[123] Al-Zeer M. A., Al-Younes H. M., Lauster D., Abu Lubad M., Meyer T. F. (2013). Autophagy restricts *Chlamydia trachomatis* growth in human macrophages via IFNG-inducible guanylate binding proteins. *Autophagy*.

[124] Owen K. A., Anderson C. J., Casanova J. E. (2016). *Salmonella* suppresses the TRIF-dependent type I interferon response in macrophages. *MBio*.

[125] Kim B.-H., Shenoy A. R., Kumar P., Das R., Tiwari S., MacMicking J. D. (2011). A family of IFN-*γ*-inducible 65-kD GTPases protects against bacterial infection. *Science*.

[126] Dawson R., Condos R., Tse D. (2009). Immunomodulation with recombinant interferon-*γ*1b in pulmonary tuberculosis. *PLoS One*.

[127] Einarsdottir T., Lockhart E., Flynn J. L. (2009). Cytotoxicity and secretion of gamma interferon are carried out by distinct CD8 T cells during *Mycobacterium tuberculosis* infection. *Infection and Immunity*.

[128] Lampe M. F., Wilson C. B., Bevan M. J., Starnbach M. N. (1998). Gamma interferon production by cytotoxic T lymphocytes is required for resolution of *Chlamydia trachomatis* infection. *Infection and Immunity*.

[129] Berg R. E., Crossley E., Murray S., Forman J. (2005). Relative contributions of NK and CD8 T cells to IFN-*γ* mediated innate immune protection against *Listeria monocytogenes*. *The Journal of Immunology*.

[130] Meek S. M., Williams M. A. (2018). IFN-gamma-dependent and independent mechanisms of CD4^+^ memory T cell-mediated protection from *Listeria* infection. *Pathogens*.

[131] Gallegos A. M., van Heijst J. W. J., Samstein M., Su X., Pamer E. G., Glickman M. S. (2011). A gamma interferon independent mechanism of CD4 T cell mediated control of *M. tuberculosis* infection in vivo. *PLoS Pathogens*.

[132] Kagina B. M. N., Abel B., Scriba T. J. (2010). Specific T cell frequency and cytokine expression profile do not correlate with protection against tuberculosis after bacillus Calmette-Guérin vaccination of newborns. *American Journal of Respiratory and Critical Care Medicine*.

[133] Sada-Ovalle I., Chiba A., Gonzales A., Brenner M. B., Behar S. M. (2008). Innate invariant NKT cells recognize *Mycobacterium tuberculosis*–infected macrophages, produce interferon-*γ*, and kill intracellular bacteria. *PLoS Pathogens*.

[134] Kaneko H., Yamada H., Mizuno S. (1999). Role of tumor necrosis factor-*α* in *Mycobacterium*-induced granuloma formation in tumor necrosis factor-alpha-deficient mice. *Laboratory Investigation*.

[135] Butler N. S., Schmidt N. W., Harty J. T. (2010). Differential effector pathways regulate memory CD8 T cell immunity against *Plasmodium berghei* versus *P. yoelii* sporozoites. *The Journal of Immunology*.

[136] Belnoue E., Costa F. T. M., Frankenberg T. (2004). Protective T cell immunity against malaria liver stage after vaccination with live sporozoites under chloroquine treatment. *The Journal of Immunology*.

[137] Robinson M. W., O’Brien R., Mackintosh C. G., Clark R. G., Griffin J. F. T. (2011). Immunoregulatory cytokines are associated with protection from immunopathology following *Mycobacterium avium* subspecies *paratuberculosis* infection in red deer. *Infection and Immunity*.

[138] Cheng W., Shivshankar P., Zhong Y., Chen D., Li Z., Zhong G. (2008). Intracellular interleukin-1*α* mediates interleukin-8 production induced by *Chlamydia trachomatis* infection via a mechanism independent of type I interleukin-1 receptor. *Infection and Immunity*.

[139] Kautz-Neu K., Kostka S. L., Dinges S., Iwakura Y., Udey M. C., von Stebut E. (2011). IL-1 signalling is dispensable for protective immunity in *Leishmania*-resistant mice. *Experimental Dermatology*.

[140] Dewamitta S. R., Nomura T., Kawamura I. (2010). Listeriolysin O-dependent bacterial entry into the cytoplasm is required for calpain activation and interleukin-1*α* secretion in macrophages infected with *Listeria monocytogenes*. *Infection and Immunity*.

[141] Behbahani H., Walther-Jallow L., Klareskog E. (2007). Proinflammatory and type 1 cytokine expression in cervical mucosa during HIV-1 and human papillomavirus infection. *JAIDS Journal of Acquired Immune Deficiency Syndromes*.

[142] Al-Attiyah R., El-Shazly A., Mustafa A. S. (2012). Comparative analysis of spontaneous and mycobacterial antigen-induced secretion of Th1, Th2 and pro-inflammatory cytokines by peripheral blood mononuclear cells of tuberculosis patients. *Scandinavian Journal of Immunology*.

[143] Umeshappa C. S., Xie Y., Xu S. (2013). Th cells promote CTL survival and memory via acquired pMHC-I and endogenous IL-2 and CD40L signaling and by modulating apoptosis-controlling pathways. *PLoS One*.

[144] Benson A., Murray S., Divakar P. (2012). Microbial infection-induced expansion of effector T cells overcomes the suppressive effects of regulatory T cells via an IL-2 deprivation mechanism. *The Journal of Immunology*.

[145] Maxwell J. R., Yadav R., Rossi R. J. (2006). IL-18 bridges innate and adaptive immunity through IFN-*γ* and the CD134 pathway. *The Journal of Immunology*.

[146] Broz P., Monack D. M. (2011). Molecular mechanisms of inflammasome activation during microbial infections. *Immunological Reviews*.

[147] Martinon F., Burns K., Tschopp J. (2002). The inflammasome: a molecular platform triggering activation of inflammatory caspases and processing of proIL-*β*. *Molecular Cell*.

[148] Goncalves-de-Albuquerque S. C., Pessoa E. S. R., Trajano-Silva L. A. M. (2017). The equivocal role of Th17 cells and neutrophils on immunopathogenesis of leishmaniasis. *Frontiers in Immunology*.

[149] Wu W., Li J., Chen F., Zhu H., Peng G., Chen Z. (2010). Circulating Th17 cells frequency is associated with the disease progression in HBV infected patients. *Journal of Gastroenterology and Hepatology*.

[150] Hartigan-O’Connor D. J., Hirao L. A., McCune J. M., Dandekar S. (2011). Th17 cells and regulatory T cells in elite control over HIV and SIV. *Current Opinion in HIV and AIDS*.

[151] Ghoreschi K., Laurence A., Yang X. P. (2010). Generation of pathogenic T_H_17 cells in the absence of TGF-*β* signalling. *Nature*.

[152] Lin Y., Ritchea S., Logar A. (2009). Interleukin-17 is required for T helper 1 cell immunity and host resistance to the intracellular pathogen *Francisella tularensis*. *Immunity*.

[153] Hamada S., Umemura M., Shiono T. (2008). IL-17A produced by *γδ* T cells plays a critical role in innate immunity against *listeria monocytogenes* infection in the liver. *The Journal of Immunology*.

[154] Foster R. G., Golden-Mason L., Rutebemberwa A., Rosen H. R. (2012). Interleukin (IL)-17/IL-22-producing T cells enriched within the liver of patients with chronic hepatitis C viral (HCV) infection. *Digestive Diseases and Sciences*.

[155] Kuchroo V. K., Anderson A. C., Petrovas C. (2014). Coinhibitory receptors and CD8 T cell exhaustion in chronic infections. *Current Opinion in HIV and AIDS*.

[156] Mueller S. N., Ahmed R. (2009). High antigen levels are the cause of T cell exhaustion during chronic viral infection. *Proceedings of the National Academy of Sciences of the United States of America*.

[157] Wang X., Claflin J., Kang H., Suzuki Y. (2005). Importance of CD8^+^V*β*8^+^ T cells in IFN-*γ*-mediated prevention of toxoplasmic encephalitis in genetically resistant BALB/c mice. *Journal of Interferon & Cytokine Research*.

[158] Kirimanjeswara G. S., Olmos S., Bakshi C. S., Metzger D. W. (2008). Humoral and cell-mediated immunity to the intracellular pathogen *Francisella tularensis*. *Immunological Reviews*.

[159] Quaresma J. A. S., Esteves P. C., de Sousa Aarão T. L., de Sousa J. R., da Silva Pinto D., Fuzii H. T. (2014). Apoptotic activity and Treg cells in tissue lesions of patients with leprosy. *Microbial Pathogenesis*.

[160] Boyer O., Saadoun D., Abriol J. (2004). CD4^+^CD25^+^ regulatory T-cell deficiency in patients with hepatitis C-mixed cryoglobulinemia vasculitis. *Blood*.

[161] Aandahl E. M., Michaelsson J., Moretto W. J., Hecht F. M., Nixon D. F. (2004). Human CD4^+^ CD25^+^ regulatory T cells control T-cell responses to human immunodeficiency virus and cytomegalovirus antigens. *Journal of Virology*.

[162] Kabelitz D. (2011). *γδ* T-cells: cross-talk between innate and adaptive immunity. *Cellular and Molecular Life Sciences*.

[163] Carding S. R., Allan W., Kyes S., Hayday A., Bottomly K., Doherty P. C. (1990). Late dominance of the inflammatory process in murine influenza by *γ*/*δ*^+^ T cells. *Journal of Experimental Medicine*.

[164] Sandor M., Sperling A. I., Cook G. A., Weinstock J. V., Lynch R. G., Bluestone J. A. (1995). Two waves of gamma delta T cells expressing different V delta genes are recruited into schistosome-induced liver granulomas. *The Journal of Immunology*.

[165] Vivier E., Tomasello E., Baratin M., Walzer T., Ugolini S. (2008). Functions of natural killer cells. *Nature Immunology*.

[166] Mavilio D., Lombardo G., Benjamin J. (2005). Characterization of CD56^−^/CD16^+^ natural killer (NK) cells: a highly dysfunctional NK subset expanded in HIV-infected viremic individuals. *Proceedings of the National Academy of Sciences of the United States of America*.

[167] Korbel D. S., Finney O. C., Riley E. M. (2004). Natural killer cells and innate immunity to protozoan pathogens. *International Journal for Parasitology*.

[168] Lewinsohn D. A., Gold M. C., Lewinsohn D. M. (2011). Views of immunology: effector T cells. *Immunological Reviews*.

[169] Malka-Ruimy C., Ben Youssef G., Lambert M. (2019). Mucosal-associated invariant T cell levels are reduced in the peripheral blood and lungs of children with active pulmonary tuberculosis. *Frontiers in Immunology*.

[170] Leeansyah E., Ganesh A., Quigley M. F. (2013). Activation, exhaustion, and persistent decline of the antimicrobial MR1-restricted MAIT-cell population in chronic HIV-1 infection. *Blood*.

[171] Yong Y. K., Saeidi A., Tan H. Y. (2018). Hyper-expression of PD-1 is associated with the levels of exhausted and dysfunctional phenotypes of circulating CD161^++^TCR iV*α*7.2^+^ mucosal-associated invariant T cells in chronic hepatitis B virus infection. *Frontiers in Immunology*.

[172] Cosgrove C., Ussher J. E., Rauch A. (2013). Early and nonreversible decrease of CD161^++^ /MAIT cells in HIV infection. *Blood*.

[173] Bitsaktsis C., Nandi B., Racine R., MacNamara K. C., Winslow G. (2007). T-cell-independent humoral immunity is sufficient for protection against fatal intracellular ehrlichia infection. *Infection and Immunity*.

[174] Hangartner L., Zinkernagel R. M., Hengartner H. (2006). Antiviral antibody responses: the two extremes of a wide spectrum. *Nature Reviews Immunolog*.

[175] Plotkin S. A. (2008). Vaccines: correlates of vaccine-induced immunity. *Clinical Infectious Diseases*.

[176] Casadevall A., Pirofski L. A. (2011). A new synthesis for antibody-mediated immunity. *Nature Immunology*.

[177] Edelson B. T., Unanue E. R. (2001). Intracellular antibody neutralizes *Listeria* growth. *Immunity*.

[178] Jayasekera J. P., Moseman E. A., Carroll M. C. (2007). Natural antibody and complement mediate neutralization of influenza virus in the absence of prior immunity. *Journal of Virology*.

[179] Nimmerjahn F., Ravetch J. V. (2011). Fc*γ*Rs in health and disease. *Current Topics in Microbiology and Immunology*.

[180] Aribam S. D., Harada T., Elsheimer-Matulova M. (2016). Specific monoclonal antibody overcomes the *Salmonella enterica* serovar Typhimurium’s adaptive mechanisms of intramacrophage survival and replication. *PLoS One*.

[181] Byndloss M. X., Tsolis R. M. (2016). Chronic bacterial pathogens: mechanisms of persistence. *Microbiology Spectrum*.

[182] Tascon R. E., Stavropoulos E., Lukacs K. V., Colston M. J. (1998). Protection against *Mycobacterium tuberculosis* infection by CD8^+^ T cells requires the production of gamma interferon. *Infection and Immunity*.

[183] Vergne I., Chua J., Lee H. H., Lucas M., Belisle J., Deretic V. (2005). Mechanism of phagolysosome biogenesis block by viable *Mycobacterium tuberculosis*. *Proceedings of the National Academy of Sciences of the United States of America*.

[184] Monack D. M. (2012). *Salmonella* persistence and transmission strategies. *Current Opinion in Microbiology*.

[185] Pizza M., Rappuoli R. (2015). Neisseria meningitidis: pathogenesis and immunity. *Current Opinion in Microbiology*.

[186] Fernández P. A., Velásquez F., Garcias-Papayani H. (2018). Fnr and ArcA regulate lipid a hydroxylation in *Salmonella* enteritidis by controlling *lpxO* expression in response to oxygen availability. *Frontiers in Microbiology*.

[187] Ratet G., Santecchia I., Fanton d’Andon M. (2017). LipL21 lipoprotein binding to peptidoglycan enables *Leptospira interrogans* to escape NOD1 and NOD2 recognition. *PLoS Pathogens*.

[188] Otten C., Brilli M., Vollmer W., Viollier P. H., Salje J. (2018). Peptidoglycan in obligate intracellular bacteria. *Molecular Microbiology*.

[189] Hernaez B., Alcami A. (2018). New insights into the immunomodulatory properties of poxvirus cytokine decoy receptors at the cell surface. *F1000Research*.

[190] Montaner S., Kufareva I., Abagyan R., Gutkind J. S. (2013). Molecular mechanisms deployed by virally encoded G protein-coupled receptors in human diseases. *Annual Review of Pharmacology and Toxicology*.

[191] Groschel M. I., Sayes F., Simeone R., Majlessi L., Brosch R. (2016). ESX secretion systems: mycobacterial evolution to counter host immunity. *Nature Reviews Microbiology*.

[192] Kim S. I., Kim S., Kim E., Hwang S. Y., Yoon H. (2018). Secretion of *Salmonella* pathogenicity island 1-encoded type III secretion system effectors by outer membrane vesicles in *Salmonella enterica* serovar typhimurium. *Frontiers in Microbiology*.

[193] Ke Y., Wang Y., Li W., Chen Z. (2015). Type IV secretion system of *Brucella* spp. and its effectors. *Frontiers in Cellular and Infection Microbiology*.

[194] Anderson M., Chen Y. H., Butler E. K., Missiakas D. M. (2011). EsaD, a secretion factor for the Ess pathway in *Staphylococcus aureus*. *Journal of Bacteriology*.

[195] Huppert L. A., Ramsdell T. L., Chase M. R., Sarracino D. A., Fortune S. M., Burton B. M. (2014). The ESX system in *Bacillus subtilis* mediates protein secretion. *PLoS One*.

[196] Petrova V. N., Russell C. A. (2018). The evolution of seasonal influenza viruses. *Nature Reviews Microbiology*.

[197] Obergfell K. P., Seifert H. S. (2015). Mobile DNA in the pathogenic *Neisseria*. *Microbiology Spectrum*.

[198] Sadarangani M., Pollard A. J., Gray-Owen S. D. (2011). Opa proteins and CEACAMs: pathways of immune engagement for pathogenic *Neisseria*. *FEMS Microbiology Reviews*.

[199] Deitsch K. W., Dzikowski R. (2017). Variant gene expression and antigenic variation by malaria parasites. *Annual Review of Microbiology*.

[200] Branchu P., Bawn M., Kingsley R. A. (2018). Genome variation and molecular epidemiology of *Salmonella enterica* serovar typhimurium pathovariants. *Infection and Immunity*.

[201] Mugnier M. R., Cross G. A. M., Papavasiliou F. N. (2015). The in vivo dynamics of antigenic variation in *Trypanosoma brucei*. *Science*.

[202] Wang J. H., Pianko M. J., Ke X. (2011). Characterization of antigenic variants of hepatitis C virus in immune evasion. *Virology Journal*.

[203] Smyth R. P., Negroni M. (2016). A step forward understanding HIV-1 diversity. *Retrovirology*.

[204] Saha A., Robertson E. S. (2011). Epstein-Barr virus–associated B-cell lymphomas: pathogenesis and clinical outcomes. *Clinical Cancer Research*.

[205] van Lint A. L., Murawski M. R., Goodbody R. E. (2010). Herpes simplex virus immediate-early ICP0 protein inhibits Toll-like receptor 2-dependent inflammatory responses and NF-*κ*B signaling. *Journal of Virology*.

[206] Bogdan C., Rollinghoff M. (1998). The immune response to *Leishmania*: mechanisms of parasite control and evasion. *International Journal for Parasitology*.

[207] Ottenhoff T. H. M., Elferink D. G., Klatser P. R., de Vries R. R. P. (1986). Cloned suppressor T cells from a lepromatous leprosy patient suppress *Mycobacterium leprae* reactive helper T cells. *Nature*.

[208] Le Negrate G., Krieg A., Faustin B. (2008). ChlaDub1 of *Chlamydia trachomatis* suppresses NF-*κ*B activation and inhibits I*κ*B*α* ubiquitination and degradation. *Cellular Microbiology*.

[209] el-Sagaff S., Salem H. S., Nichols W., Tonkel A. K., bo-Zenadah N. Y. (2005). Cell death pattern in cerebellum neurons infected with *Toxoplasma gondii*. *Journal of the Egyptian Society of Parasitology*.

[210] Reichelt M., Zerboni L., Arvin A. M. (2008). Mechanisms of varicella-zoster virus neuropathogenesis in human dorsal root ganglia. *Journal of Virology*.

[211] Baldwin C. L., Goenka R. (2006). Host immune responses to the intracellular bacteria *Brucella*: does the bacteria instruct the host to facilitate chronic infection?. *Critical Reviews in Immunology*.

[212] Rajeeve K., Das S., Prusty B. K., Rudel T. (2018). *Chlamydia trachomatis* paralyses neutrophils to evade the host innate immune response. *Nature Microbiology*.

[213] Zhong G. (2009). Killing me softly: chlamydial use of proteolysis for evading host defenses. *Trends in Microbiology*.

[214] Brodmann M., Dreier R. F., Broz P., Basler M. (2017). *Francisella* requires dynamic type VI secretion system and ClpB to deliver effectors for phagosomal escape. *Nature Communications*.

[215] Niu H., Xiong Q., Yamamoto A., Hayashi-Nishino M., Rikihisa Y. (2012). Autophagosomes induced by a bacterial Beclin 1 binding protein facilitate obligatory intracellular infection. *Proceedings of the National Academy of Sciences of the United States of America*.

[216] Luo Z. Q. (2012). Legionella secreted effectors and innate immune responses. *Cellular Microbiology*.

[217] Poussin M. A., Goldfine H. (2010). Evidence for the involvement of ActA in maturation of the *Listeria monocytogenes* phagosome. *Cell Research*.

[218] Sansonetti P. J. (2004). War and peace at mucosal surfaces. *Nature Reviews Immunology*.

[219] Ye Z., Petrof E. O., Boone D., Claud E. C., Sun J. (2007). *Salmonella* effector AvrA regulation of colonic epithelial cell inflammation by deubiquitination. *The American Journal of Pathology*.

[220] Cherayil B. J., McCormick B. A., Bosley J. (2000). *Salmonella enterica* serovar typhimurium-dependent regulation of inducible nitric oxide synthase expression in macrophages by invasins SipB, SipC, and SipD and effector SopE2. *Infection and Immunity*.

[221] Molestina R. E., Sinai A. P. (2005). Detection of a novel parasite kinase activity at the *Toxoplasma gondii* parasitophorous vacuole membrane capable of phosphorylating host I*κ*B*α*. *Cellular Microbiology*.

[222] Popescu I., Macedo C., Abu-Elmagd K. (2007). EBV-specific CD8^+^ T cell reactivation in transplant patients results in expansion of CD8^+^ type-1 regulatory T cells. *American Journal of Transplantation*.

[223] Vanderplasschen A., Mathew E., Hollinshead M., Sim R. B., Smith G. L. (1998). Extracellular enveloped vaccinia virus is resistant to complement because of incorporation of host complement control proteins into its envelope. *Proceedings of the National Academy of Sciences of the United States of America*.

[224] Matsui M., Moriya O., Yoshimoto T., Akatsuka T. (2005). T-bet is required for protection against vaccinia virus infection. *Journal of Virology*.

[225] Kohyama S., Ohno S., Isoda A. (2007). IL-23 enhances host defense against vaccinia virus infection via a mechanism partly involving IL-17. *The Journal of Immunology*.

[226] Bowen D. G., Walker C. M. (2005). Adaptive immune responses in acute and chronic hepatitis C virus infection. *Nature*.

[227] Alatrakchi N., Graham C. S., van der Vliet H. J. J., Sherman K. E., Exley M. A., Koziel M. J. (2007). Hepatitis C virus (HCV)-specific CD8^+^ cells produce transforming growth factor *β* that can suppress HCV-specific T-cell responses. *Journal of Virology*.

[228] Yao Z. Q., Ray S., Eisen-Vandervelde A., Waggoner S., Hahn Y. S. (2001). Hepatitis C virus: immunosuppression by complement regulatory pathway. *Viral Immunology*.

[229] de Souza Sales J., Lara F. A., Amadeu T. P. (2011). The role of indoleamine 2, 3-dioxygenase in lepromatous leprosy immunosuppression. *Clinical & Experimental Immunology*.

[230] Belkaid Y., Piccirillo C. A., Mendez S., Shevach E. M., Sacks D. L. (2002). CD4^+^CD25^+^ regulatory T cells control *Leishmania major* persistence and immunity. *Nature*.

[231] Scott-Browne J. P., Shafiani S., Tucker-Heard G. (2007). Expansion and function of Foxp3-expressing T regulatory cells during tuberculosis. *Journal of Experimental Medicine*.

[232] Walther M., Tongren J. E., Andrews L. (2005). Upregulation of TGF-*β*, *FOXP3*, and CD4^+^CD25^+^ regulatory T cells correlates with more rapid parasite growth in human malaria infection. *Immunity*.

[233] Mota L. J., Cornelis G. R. (2005). The bacterial injection kit: type III secretion systems. *Annals of Medicine*.

[234] Christie P. J., Atmakuri K., Krishnamoorthy V., Jakubowski S., Cascales E. (2005). Biogenesis, architecture, and function of bacterial type IV secretion systems. *Annual Review of Microbiology*.

[235] Badr G., Borhis G., Treton D., Moog C., Garraud O., Richard Y. (2005). HIV type 1 glycoprotein 120 inhibits human B cell chemotaxis to CXC chemokine ligand (CXCL) 12, CC chemokine ligand (CCL)20, and CCL21. *The Journal of Immunology*.

[236] Erridge C., Bennett-Guerrero E., Poxton I. R. (2002). Structure and function of lipopolysaccharides. *Microbes and Infection*.

[237] Zughaier S. M., Kandler J. L., Balthazar J. T., Shafer W. M. (2015). Phosphoethanolamine modification of Neisseria gonorrhoeae lipid a reduces autophagy flux in macrophages. *PLoS One*.

[238] Rosadini C. V., Zanoni I., Odendall C. (2015). A single bacterial immune evasion strategy dismantles both MyD88 and TRIF signaling pathways downstream of TLR4. *Cell Host & Microbe*.

[239] Byndloss M. X., Rivera-Chavez F., Tsolis R. M., Baumler A. J. (2017). How bacterial pathogens use type III and type IV secretion systems to facilitate their transmission. *Current Opinion in Microbiology*.

[240] Heidarieh H., Hernaez B., Alcami A. (2015). Immune modulation by virus-encoded secreted chemokine binding proteins. *Virus Research*.

[241] Ojha H., Panwar H. S., Gorham R. D., Morikis D., Sahu A. (2014). Viral regulators of complement activation: structure, function and evolution. *Molecular Immunology*.

[242] Zheng D., Chen H., Bartee M. Y. (2012). Virus-derived anti-inflammatory proteins: potential therapeutics for cancer. *Trends in Molecular Medicine*.

[243] Zhou P., Zeng W., Zhang X., Li S. (2017). The genetic evolution of canine parvovirus - a new perspective. *PLoS One*.

[244] Kurt-Jones E. A., Orzalli M. H., Knipe D. M., Osterrieder K. Innate immune mechanisms and herpes simplex virus infection and disease. *Cell Biology of Herpes Viruses. Advances in Anatomy, Embryology and Cell Biology, Vol 223*.

[245] Bogdan C. (2008). Mechanisms and consequences of persistence of intracellular pathogens: leishmaniasis as an example. *Cellular Microbiology*.

[246] Viboud G. I., Bliska J. B. (2005). *Yersinia* outer proteins: role in modulation of host cell signaling responses and pathogenesis. *Annual Review of Microbiology*.

[247] Dortet L., Mostowy S., Louaka A. S. (2011). Recruitment of the major vault protein by InlK: a *Listeria monocytogenes* strategy to avoid autophagy. *PLoS Pathogens*.

[248] Nguyen B. N., Peterson B. N., Portnoy D. A. (2019). Listeriolysin O: a phagosome-specific cytolysin revisited. *Cellular Microbiology*.

[249] Uchiya K., Barbieri M. A., Funato K., Shah A. H., Stahl P. D., Groisman E. A. (1999). A *Salmonella* virulence protein that inhibits cellular trafficking. *The EMBO Journal*.

[250] Hu D., Wu J., Wang W. (2015). Autophagy regulation revealed by SapM-induced block of autophagosome-lysosome fusion via binding RAB7. *Biochemical and Biophysical Research Communications*.

[251] Miller S. I., Mekalanos J. J. (1990). Constitutive expression of the phoP regulon attenuates *Salmonella* virulence and survival within macrophages. *Journal of Bacteriology*.

[252] Howe D., Mallavia L. P. (2000). *Coxiella burnetii* exhibits morphological change and delays phagolysosomal fusion after internalization by J774A.1 cells. *Infection and Immunity*.

[253] Alexander J., Satoskar A. R., Russell D. G. (1999). *Leishmania* species: models of intracellular parasitism. *Journal of Cell Science*.

[254] Fortier A. H., Leiby D. A., Narayanan R. B. (1995). Growth of *Francisella tularensis* LVS in macrophages: the acidic intracellular compartment provides essential iron required for growth. *Infection and Immunity*.

[255] Tranchemontagne Z. R., Camire R. B., O'Donnell V. J., Baugh J., Burkholder K. M. (2016). *Staphylococcus aureus* strain USA300 perturbs acquisition of lysosomal enzymes and requires phagosomal acidification for survival inside macrophages. *Infection and Immunity*.

[256] de Jong N. W. M., Ramyar K. X., Guerra F. E. (2017). Immune evasion by a staphylococcal inhibitor of myeloperoxidase. *Proceedings of the National Academy of Sciences of the United States of America*.

[257] Rahman M. M., McFadden G. (2011). Modulation of NF-*κ*B signalling by microbial pathogens. *Nature Reviews Microbiology*.

[258] Mukherjee S., Keitany G., Li Y. (2006). *Yersinia* YopJ acetylates and inhibits kinase activation by blocking phosphorylation. *Science*.

[259] le Negrate G., Faustin B., Welsh K. (2008). *Salmonella* secreted factor L deubiquitinase of *Salmonella typhimurium* inhibits NF-*κ*B, suppresses I*κ*B*α* ubiquitination and modulates innate immune responses. *The Journal of Immunology*.

[260] Wolf K., Plano G. V., Fields K. A. (2009). A protein secreted by the respiratory pathogen *Chlamydia pneumoniae* impairs IL-17 signalling via interaction with human Act1. *Cellular Microbiology*.

[261] Harte M. T., Haga I. R., Maloney G. (2003). The poxvirus protein A52R targets Toll-like receptor signaling complexes to suppress host defense. *Journal of Experimental Medicine*.

[262] Breiman A., Grandvaux N., Lin R. (2005). Inhibition of RIG-I-dependent signaling to the interferon pathway during hepatitis C virus expression and restoration of signaling by IKK*ε*. *Journal of Virology*.

[263] Carty M., Bowie A. G. (2010). Recent insights into the role of Toll-like receptors in viral infection. *Clinical & Experimental Immunology*.

[264] Uchida L., Espada-Murao L. A., Takamatsu Y. (2014). The dengue virus conceals double-stranded RNA in the intracellular membrane to escape from an interferon response. *Scientific Reports*.

[265] Neufeldt C. J., Joyce M. A., van Buuren N. (2016). The hepatitis C virus-induced membranous web and associated nuclear transport machinery limit access of pattern recognition receptors to viral replication sites. *PLoS Pathogens*.

[266] Feng Q., Langereis M. A., Lork M. (2014). Enterovirus 2Apro targets MDA5 and MAVS in infected cells. *Journal of Virology*.

[267] Rajsbaum R., Albrecht R. A., Wang M. K. (2012). Species-specific inhibition of RIG-I ubiquitination and IFN induction by the influenza A virus NS1 protein. *PLoS Pathogens*.

[268] Orzalli M. H., DeLuca N. A., Knipe D. M. (2012). Nuclear IFI16 induction of IRF-3 signaling during herpesviral infection and degradation of IFI16 by the viral ICP0 protein. *Proceedings of the National Academy of Sciences of the United States of America*.

[269] Lahaye X., Satoh T., Gentili M. (2013). The capsids of HIV-1 and HIV-2 determine immune detection of the viral cDNA by the innate sensor cGAS in dendritic cells. *Immunity*.

[270] Chan Y. K., Gack M. U. (2016). Viral evasion of intracellular DNA and RNA sensing. *Nature Reviews Microbiology*.

[271] DePaolo R. W., Tang F., Kim I. Y. (2008). Toll-like receptor 6 drives differentiation of tolerogenic dendritic cells and contributes to LcrV-mediated plague pathogenesis. *Cell Host & Microbe*.

[272] Paquette N., Conlon J., Sweet C. (2012). Serine/threonine acetylation of TGF*β*-activated kinase (TAK1) by Yersinia pestis YopJ inhibits innate immune signaling. *Proceedings of the National Academy of Sciences of the United States of America*.

[273] Wu H., Jones R. M., Neish A. S. (2012). The *Salmonella* effector AvrA mediates bacterial intracellular survival during infection *in vivo*. *Cellular Microbiology*.

[274] Van Avondt K., van Sorge N. M., Meyaard L. (2015). Bacterial immune evasion through manipulation of host inhibitory immune signaling. *PLoS Pathogens*.

[275] Nakayama M., Kurokawa K., Nakamura K. (2012). Inhibitory receptor paired Ig-like receptor B is exploited by *Staphylococcus aureus* for virulence. *The Journal of Immunology*.

[276] Wang Y. C., Chen C. L., Sheu B. S. (2014). *Helicobacter pylori* infection activates Src homology-2 domain-containing phosphatase 2 to suppress IFN-*γ* signaling. *The Journal of Immunology*.

[277] Choy A., Dancourt J., Mugo B. (2012). The *Legionella* effector RavZ inhibits host autophagy through irreversible Atg8 deconjugation. *Science*.

[278] Sparrer K. M. J., Gableske S., Zurenski M. A. (2017). TRIM23 mediates virus-induced autophagy via activation of TBK1. *Nature Microbiology*.

[279] Richards A. L., Soares-Martins J. A. P., Riddell G. T., Jackson W. T. (2014). Generation of unique poliovirus RNA replication organelles. *MBio*.

[280] Hansen M. D., Johnsen I. B., Stiberg K. A. (2017). Hepatitis C virus triggers Golgi fragmentation and autophagy through the immunity-related GTPase M. *Proceedings of the National Academy of Sciences of the United States of America*.

[281] Leymarie O., Lepont L., Berlioz-Torrent C. (2017). Canonical and non-canonical autophagy in HIV-1 replication cycle. *Viruses*.

[282] Montefiori D. C., Cornell R. J., Zhou J. Y., Zhou J. T., Hirsch V. M., Johnson P. R. (1994). Complement control proteins, CD46, CD55, and CD59, as common surface constituents of human and simian immunodeficiency viruses and possible targets for vaccine protection. *Virology*.

[283] Blom A. M. (2004). Strategies developed by bacteria and virus for protection from the human complement system. *Scandinavian Journal of Clinical and Laboratory Investigation*.

[284] Ram S., Cullinane M., Blom A. M. (2001). Binding of C4b-binding protein to porin: a molecular mechanism of serum resistance of *Neisseria gonorrhoeae*. *Journal of Experimental Medicine*.

[285] Alcami A., Koszinowski U. H. (2000). Viral mechanisms of immune evasion. *Immunology Today*.

[286] Alcami A. (2003). Viral mimicry of cytokines, chemokines and their receptors. *Nature Reviews Immunology*.

[287] Beuscher H. U., Rodel F., Forsberg A., Rollinghoff M. (1995). Bacterial evasion of host immune defense: *Yersinia enterocolitica* encodes a suppressor for tumor necrosis factor alpha expression. *Infection and Immunity*.

[288] Caron E., Gross A., Liautard J. P., Dornand J. (1996). *Brucella* species release a specific, protease-sensitive, inhibitor of TNF-alpha expression, active on human macrophage-like cells. *The Journal of Immunology*.

[289] Matsui K. (1996). A purified protein from *Salmonella typhimurium* inhibits proliferation of murine splenic anti-CD3 antibody-activated T-lymphocytes. *FEMS Immunology & Medical Microbiology*.

[290] Mintz C. S., Miller R. D., Gutgsell N. S., Malek T. (1993). *Legionella pneumophila* protease inactivates interleukin-2 and cleaves CD4 on human T cells. *Infection and Immunity*.

[291] Lodoen M. B., Lanier L. L. (2005). Viral modulation of NK cell immunity. *Nature Reviews Microbiology*.

[292] Ju Y., Hou N., Meng J. (2010). T cell immunoglobulin- and mucin-domain-containing molecule-3 (Tim-3) mediates natural killer cell suppression in chronic hepatitis B. *Journal of Hepatology*.

[293] Ströh L. J., Nagarathinam K., Krey T. (2018). Conformational flexibility in the CD81-binding site of the hepatitis C virus glycoprotein E2. *Frontiers in Immunology*.

[294] Rappocciolo G., Jais M., Piazza P. A., DeLucia D. C., Jenkins F. J., Rinaldo C. R. (2017). Human herpesvirus 8 infects and replicates in Langerhans cells and interstitial dermal dendritic cells and impairs their function. *Journal of Virology*.

[295] Gondar V., Molina-Jiménez F., Hishiki T. (2015). Apolipoprotein E, but not apolipoprotein B, is essential for efficient cell-to-cell transmission of hepatitis C virus. *Journal of Virology*.

[296] Li Y., Pierce B. G., Wang Q. (2015). Structural basis for penetration of the glycan shield of hepatitis C virus E2 glycoprotein by a broadly neutralizing human antibody. *Journal of Biological Chemistry*.

[297] Seitz H., Schmitt M., Bohmer G., Kopp-Schneider A., Muller M. (2013). Natural variants in the major neutralizing epitope of human papillomavirus minor capsid protein L2. *International Journal of Cancer*.

[298] Boulton I. C., Gray-Owen S. D. (2002). Neisserial binding to CEACAM1 arrests the activation and proliferation of CD4^+^ T lymphocytes. *Nature Immunology*.

[299] Sewald X., Jiménez-Soto L., Haas R. (2011). PKC-dependent endocytosis of the *Helicobacter pylori* vacuolating cytotoxin in primary T lymphocytes. *Cellular Microbiology*.

[300] Srivastava S., Grace P. S., Ernst J. D. (2016). Antigen export reduces antigen presentation and limits T cell control of *M. tuberculosis*. *Cell Host & Microbe*.

[301] Velásquez L. N., Milillo M. A., Delpino M. V. (2017). *Brucella abortus* down-regulates MHC class II by the IL-6-dependent inhibition of CIITA through the downmodulation of IFN regulatory factor-1 (IRF-1). *Journal of Leukocyte Biology*.

[302] Besbes A., le Goff S., Antunes A. (2015). Hyperinvasive meningococci induce intra-nuclear cleavage of the NF-*κ*B protein p65/RelA by meningococcal IgA protease. *PLoS Pathogens*.

[303] Havel J. J., Chowell D., Chan T. A. (2019). The evolving landscape of biomarkers for checkpoint inhibitor immunotherapy. *Nature Reviews Cancer*.

[304] Wang J., Roderiquez G., Norcross M. A. (2012). Control of adaptive immune responses by *Staphylococcus aureus* through IL-10, PD-L1, and TLR2. *Scientific Reports*.

[305] Hou N., Zou Y., Piao X. (2016). T-cell immunoglobulin- and mucin-domain-containing molecule 3 signaling blockade improves cell-mediated immunity against malaria. *Journal of Infectious Diseases*.

[306] Jayaraman P., Jacques M. K., Zhu C. (2016). TIM3 mediates T cell exhaustion during *Mycobacterium tuberculosis* infection. *PLoS Pathogens*.

[307] Wykes M. N., Lewin S. R. (2018). Immune checkpoint blockade in infectious diseases. *Nature Reviews Immunology*.

[308] Stephenson H. N., Herzig A., Zychlinsky A. (2016). Beyond the grave: when is cell death critical for immunity to infection?. *Current Opinion in Immunology*.

[309] Carpenter D., Hsiang C., Jiang X. (2015). The herpes simplex virus type 1 (HSV-1) latency-associated transcript (LAT) protects cells against cold-shock-induced apoptosis by maintaining phosphorylation of protein kinase B (AKT). *Journal of Neurovirology*.

[310] Mills S. D., Boland A., Sory M. P. (1997). *Yersinia enterocolitica* induces apoptosis in macrophages by a process requiring functional type III secretion and translocation mechanisms and involving YopP, presumably acting as an effector protein. *Proceedings of the National Academy of Sciences of the United States of America*.

[311] Knodler L. A., Finlay B. B., Steele-Mortimer O. (2005). The *Salmonella* effector protein SopB protects epithelial cells from apoptosis by sustained activation of Akt. *Journal of Biological Chemistry*.

[312] Byrne G. I., Ojcius D. M. (2004). *Chlamydia* and apoptosis: life and death decisions of an intracellular pathogen. *Nature Reviews Microbiology*.

[313] Sixt B. S., Núñez-Otero C., Kepp O., Valdivia R. H., Kroemer G. (2018). *Chlamydia trachomatis* fails to protect its growth niche against pro-apoptotic insults. *Cell Death & Differentiation*.

[314] Clifton D. R., Goss R. A., Sahni S. K. (1998). NF-*κ*B-dependent inhibition of apoptosis is essential for host cellsurvival during *Rickettsia rickettsii* infection. *Proceedings of the National Academy of Sciences of the United States of America*.

[315] Bisle S., Klingenbeck L., Borges V. (2016). The inhibition of the apoptosis pathway by the *Coxiella burnetii* effector protein CaeA requires the EK repetition motif, but is independent of survivin. *Virulence*.

[316] Tundup S., Mohareer K., Hasnain S. E. (2014). *Mycobacterium tuberculosis* PE25/PPE41 protein complex induces necrosis in macrophages: role in virulence and disease reactivation?. *FEBS Open Bio*.

[317] Orzalli M. H., Kagan J. C. (2017). Apoptosis and necroptosis as host defense strategies to prevent viral infection. *Trends in Cell Biology*.

[318] Jorgensen I., Miao E. A. (2015). Pyroptotic cell death defends against intracellular pathogens. *Immunological Reviews*.

